# Eco-Friendly Rapid-Setting Concrete Incorporating Waste-Derived Additives for Post-Disaster Reconstruction

**DOI:** 10.3390/ma19061218

**Published:** 2026-03-19

**Authors:** Anna Starczyk-Kołbyk, Waldemar Łasica, Emil Kardaszuk, Michał Gregorczyk

**Affiliations:** Research Laboratory, Faculty of Civil Engineering and Geodesy, Military University of Technology, Gen. S. Kaliskiego 2, 00-908 Warsaw, Poland; waldemar.lasica@wat.edu.pl (W.Ł.); emil.kardaszuk@wat.edu.pl (E.K.); michal.gregorczyk@wat.edu.pl (M.G.)

**Keywords:** rapid-setting concrete, eco-friendly concrete, concrete durability, rapid reconstruction, waste glass, glass aggregate, sodium silicate, hybrid aggregate skeleton

## Abstract

This study investigates an eco-friendly rapid-setting concrete developed for emergency repair and accelerated post-disaster reconstruction. The proposed material concept combines a low-emission multicomponent cement, CEM V/A (S-V) 42.5 N-LH/HSR/NA, with a hybrid aggregate skeleton composed of crushed granite and waste soda–lime glass, as well as a waste-derived silicate additive system based on aqueous sodium silicate, glass dust and glass powder. One reference mixture (R) and five modified mixtures (M1–M5) were designed to assess the effects of partial replacement of natural aggregate by glass aggregate and of the dosage of the silicate-based additive system on concrete performance. The experimental programme included setting time, compressive strength, splitting tensile strength, water absorption, freeze–thaw resistance and microstructural observations. Among the modified concretes, the mixture containing 5 vol.% glass aggregate (M1) showed the most favourable mechanical performance after 28 days, reaching a compressive strength of 95.1 ± 2.4 MPa and a splitting tensile strength of 4.82 ± 0.29 MPa, compared with 45.5 ± 0.8 MPa and 2.18 ± 0.11 MPa, respectively, for the reference concrete. Higher glass contents reduced strength relative to M1, but the modified mixtures still maintained satisfactory performance. The silicate-based system significantly affected setting behaviour; in mixture M5, the initial and final setting times were reduced from 380 ± 5 min and 497 ± 5 min to 213 ± 5 min and 307 ± 5 min, respectively. The results show that the combined use of CEM V cement, waste glass and silicate-based waste-derived additives can produce concretes with rapid-setting, high strength and satisfactory durability-related properties. The developed material may therefore be considered a promising solution for selected rapid-repair and reconstruction applications, particularly in lightly reinforced or unreinforced concrete elements requiring fast restoration of functionality.

## 1. Introduction

Natural disasters and technical failures, including floods, earthquakes, landslides and localised damage to transport and building infrastructure, often require repair and reconstruction works to be carried out under severe time constraints and demanding logistical conditions. In such circumstances, construction materials capable of rapid strength development are essential, as they allow for the early installation of structural elements and the swift restoration of functionality. Although conventional structural concrete remains one of the most widely used construction materials, it is characterised by relatively long setting and hardening periods, as well as a substantial carbon footprint arising from its high Portland clinker content and extensive consumption of natural resources.

In line with European Union climate policy and the principles of the circular economy, increasing emphasis is being placed on solutions that simultaneously reduce CO_2_ emissions and limit the depletion of natural aggregate resources. One promising approach involves the use of multicomponent cements combined with industrial by-products and recycled aggregates. Among these, waste glass has attracted considerable interest, as, following suitable processing, it can function both as an aggregate and as a pozzolanic mineral additive.

A key challenge associated with the use of glass in concrete is the potential for alkali–silica reaction (ASR), which results in the formation of expansive reaction products and progressive deterioration of the concrete microstructure. This risk can be mitigated through the careful selection of glass particle size, the use of low-alkali or pozzolanic cements, and the incorporation of reactive mineral additives capable of binding free alkalis. Concurrently, research is being conducted on modifying the rheological properties and setting time of cement pastes through the use of sodium silicate solutions and glass powder, facilitating accelerated early-age strength development without excessive heat of hydration.

The present study focuses on the development of an eco-friendly rapid-setting concrete intended for emergency repair works and accelerated reconstruction of locally damaged infrastructure components after disaster-related or accidental events. In practical terms, the targeted applications include situations requiring the rapid restoration of serviceability, such as replacement of small concrete elements, repair of damaged unreinforced or lightly reinforced members, and execution of prefabricated or cast-in-place components under severe time constraints. A low-emission CEM V/A (S-V) 42.5 N-LH/HSR/NA cement was combined with a hybrid aggregate skeleton comprising granite aggregate and soda–lime glass, supplemented by waste-derived additives in the form of glass dust and powder introduced together with sodium silicate. The primary objective of the study is to assess the influence of glass aggregate content and silicate additive dosage on the mechanical performance and durability of concrete mixtures designed to enable rapid restoration of structural functionality [1].

## 2. Literature Review

The use of recycled and waste-derived materials in concrete has received increasing attention in recent years in response to environmental regulations, decarbonisation targets and the growing pressure on natural raw materials. Among these materials, soda–lime waste glass is of particular interest because it is widely available, rich in amorphous silica and, after suitable processing, can be used either as aggregate or as a finely ground mineral component in cementitious composites [2,3,4,5,6].

Previous studies have shown that waste glass may influence concrete behaviour in different ways depending on its form, particle size, replacement level and the characteristics of the binder system. When used as aggregate, glass modifies the grading curve, particle shape and surface characteristics of the granular skeleton. At low- or moderate replacement levels, this may improve packing density and reduce internal voids, which can have a positive effect on compressive strength. However, the effect is not always beneficial. Because glass particles have a smooth surface and different stiffness than conventional mineral aggregate, they may weaken the interfacial transition zone and reduce mechanical interlock, especially at higher replacement ratios. For this reason, glass aggregate should not be treated as a simple one-to-one substitute for natural aggregate, but rather as a design variable that may improve or impair performance depending on the overall mixture composition [2,3,4,5,6].

A major concern in concretes containing waste glass is alkali–silica reaction (ASR). Since soda–lime glass contains reactive amorphous silica, it may participate in expansive reactions in an alkaline pore solution, leading to internal stresses, microcracking and long-term deterioration. The literature consistently indicates that ASR risk depends mainly on the size and amount of glass particles, the alkali content of the binder and the moisture conditions in service [7,8,9,10,11,12]. Finely ground glass may reduce expansion because its behaviour becomes more pozzolanic and less reactive in the classical ASR sense. Nevertheless, this effect cannot be assumed automatically, as the final outcome depends on the entire chemical environment of the cementitious system. The current state of knowledge therefore supports a combined mitigation strategy based on particle-size control, the use of blended or low-alkali binders and the incorporation of mineral components capable of binding alkalis [8,9,10,11,12].

Another line of research relevant to the present study concerns the use of sodium silicate and related silicate-based systems for modifying hydration kinetics and setting behaviour. These materials are used to accelerate structural build-up, influence rheology and promote early-age strength development. Their action may be particularly useful in repair materials and concretes intended for rapid construction or emergency interventions, where shortening the time required to restore serviceability is essential [13,14,15,16]. At the same time, the literature shows that the effect of sodium silicate is strongly dosage-dependent. Insufficient amounts may not provide the intended acceleration, whereas excessive amounts may lead to overly rapid stiffening, loss of workable time, shrinkage-related cracking or other adverse early-age effects. This means that the performance of silicate-based systems must be evaluated not only in terms of setting time, but also with regard to practical workability and the risk of microstructural damage [13,14,15,16].

The use of finely divided glass together with sodium silicate introduces an additional issue. Glass dust and glass powder may act not only as reactive silica sources, but also as very fine particles affecting nucleation and packing in the cement paste. This may support matrix densification and early structural development. However, because both the glass-bearing fraction and the sodium silicate contribute chemically to the system, their combined use requires careful durability assessment, particularly when the concrete is intended for moisture exposure, freeze–thaw conditions or other aggressive environments [7,8,9,10,11,12,13,14,15,16].

In parallel, current concrete design increasingly relies on particle packing concepts. Packing-based approaches are used to optimise the granular skeleton, reduce voids and lower paste demand while maintaining good mechanical performance and acceptable workability. In systems containing both mineral aggregate and recycled glass, this is particularly important, because a well-designed hybrid skeleton may partly compensate for the unfavourable surface characteristics of glass and improve the overall compactness of the composite [17,18,19]. Nevertheless, packing optimisation alone is not sufficient. The literature indicates that concretes with recycled components should be assessed as integrated systems in which aggregate grading, binder composition, additive dosage and durability requirements are considered together [17,18,19].

Durability remains a critical issue in repair concretes and materials intended for infrastructure applications. In practice, rapid setting and rapid strength development are not enough if the concrete does not provide sufficient resistance to water ingress, freezing and thawing, or other environmental actions relevant to the intended exposure class. Standards and durability-oriented studies emphasise that binder selection, pore structure, air-void characteristics and mixture proportioning should be compatible with the expected service conditions [20,21,22,23]. For this reason, the most reliable studies on rapid-setting materials do not focus only on early-age kinetics but also include durability-related verification.

Although the literature on waste glass in concrete, ASR mitigation, sodium silicate-based modifiers and packing-oriented design is already extensive, these topics are often investigated separately. Many studies deal with waste glass mainly from the perspective of durability or supplementary cementitious use, while others focus on rapid-setting systems without a broader analysis of durability-related properties. Comparatively fewer studies address these issues within one coherent material concept aimed at eco-friendly rapid-setting concrete for time-critical repair and reconstruction [7,8,9,10,11,12,13,14,15,16,17,18,19,20,21,22,23]. This is especially true for systems that combine a low-emission multicomponent cement, a hybrid glass–mineral aggregate skeleton and a waste-derived silicate-based fine additive system in one application-oriented design.

The present study was developed in response to this research gap. It examines a rapid-setting concrete concept in which waste glass is used in both aggregate and fine fractions and is combined with a sodium silicate-based system and a low-emission CEM V binder. The work therefore addresses not only setting behaviour and mechanical performance but also selected durability-related properties relevant to emergency repair and post-disaster reconstruction applications.

## 3. Aim, Scope and Novelty of the Study

The aim of this study was to develop and evaluate an eco-friendly rapid-setting concrete intended for emergency repair works and accelerated reconstruction of locally damaged infrastructure elements. The material concept was designed for applications requiring short setting time, rapid restoration of serviceability and reduced environmental burden compared with conventional concretes based exclusively on high-clinker cement and natural aggregates.

The scope of the study covered the design and laboratory verification of six concrete compositions: one reference mixture (R) and five modified mixtures (M1–M5). The investigated variables were the partial volumetric replacement of natural aggregate by soda–lime glass aggregate in the amount of 5–15 vol.% (mixtures M1–M3) and the dosage of a waste-derived sodium silicate–glass dust–glass powder system (mixtures M4 and M5). All mixtures were produced using the same low-emission multicomponent cement, CEM V/A (S-V) 42.5 N-LH/HSR/NA, and a hybrid glass–granite aggregate skeleton. The experimental programme included the assessment of selected fresh-state properties, setting time, compressive strength, splitting tensile strength, water absorption, freeze–thaw resistance and microstructural features.

The novelty of the study lies in the integration of several material and functional concepts within one application-oriented concrete system. First, the proposed solution combines a low-emission multicomponent binder with a hybrid aggregate skeleton composed of crushed granite and waste soda–lime glass. Second, waste glass was incorporated not only as aggregate, but also in finely divided form within a silicate-based additive system intended to modify setting behaviour and early microstructural development. Third, the study evaluated this combined material concept not only in terms of mechanical performance, but also with respect to durability-related properties relevant to practical repair applications under demanding exposure conditions.

An additional novel aspect is the application focus of the work. While previous studies have often analysed waste glass, silicate activators or durability issues separately, the present study addresses their combined use in a rapid-setting concrete specifically intended for time-critical repair and post-disaster reconstruction scenarios. In this sense, the work contributes an experimentally verified material concept that links sustainable material selection with functional requirements characteristic of emergency construction practice.

## 4. Methodology and Method

The experimental programme was designed to assess the influence of a hybrid glass–granite aggregate skeleton and waste-derived silicate additives on the properties of both fresh and hardened concrete. In total, five modified mixtures (M1–M5) and one reference mixture (R) were prepared, as follows:

- M1—5% by volume of glass aggregate replacing natural aggregate,
- M2—10% glass aggregate,
- M3—15% glass aggregate,
- M4—concrete modified with a sodium silicate suspension combined with glass dust and glass powder (2.5% by mass of binder),
- M5—as M4, with a dosage of 5.0% by mass of binder.

All mixtures were produced using the same low-emission multicomponent cement, CEM V/A (S-V) 42.5 N-LH/HSR/NA, and a hybrid aggregate skeleton composed of granite aggregate and soda–lime glass. The primary variables were the volumetric proportion of glass aggregate and the dosage of the polydisperse sodium silicate–glass dust–glass powder system.

The test programme comprised the following assessments:

- Fresh concrete properties: consistency (slump test), air content, and setting time (Vicat apparatus),
- Mechanical properties: compressive strength (f_ck,cube_) and splitting tensile strength (f_ct_) after 7, 28, and 56 days of curing,
- Durability-related properties: water absorption, freeze–thaw resistance, and alkali–silica reactivity,
- Microstructural analysis: scanning electron microscopy (SEM) observations and energy-dispersive X-ray spectroscopy (EDS) analysis of hydration products.

Each test series was conducted using a minimum of three specimens. The results were analysed statistically using descriptive statistics and simple regression models.

Concrete mix compositions were developed based on a multicriteria evaluation of mixture proportioning methods for hydraulic binder concretes, with the B. Bukowski method adopted as the primary design approach. This method was used to determine the quantitative composition of mixtures intended for concrete class C30/37 with a bulk density of ρ < 2600 kg/m^3^, designed for exposure classes XC3, XD2, XF3, XA2, and the additional class XM2.

The principal design assumptions were as follows:

- a minimum cement content of 320 kg/m^3^ and a water-to-cement ratio w/c ≤ 0.50,
- achievement of consistency class S1/S2 using a superplasticiser based on modified polycarboxylate ethers,
- design of hybrid aggregate skeletons comprising soda–lime glass aggregate (0/2 mm) and granite aggregate (2/8 mm) with constant bulk density,
- control of air content in fresh concrete (3.8–4.5%) and hardened concrete (4.5–5.5%).

Aggregate grading was determined using standard limiting curves for the 0/8 mm fraction, with identification of characteristic filler and sand points and selection of appropriate proportions of filler fractions and glass powder. Based on these parameters, preliminary reference mix designs were calculated using the equations of the B. Bukowski method [14], followed by quantitative adjustments to account for the target consistency and applied material modifications [24,25,26,27,28,29,30,31,32,33,34,35,36,37].

**Figure 1:**
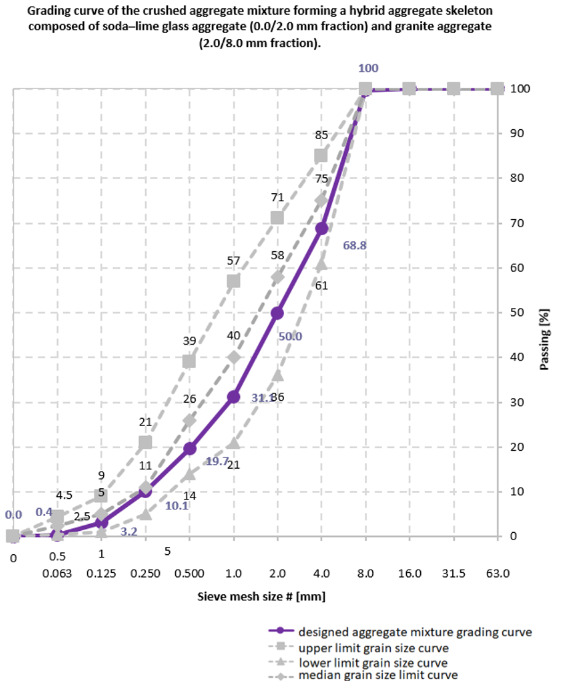
Mix compositions for determining the initial and final setting times of cement pastes, including the reference mix (R) and mixes modified with waste-derived materials (M1–M5) [own analysis].

**Figure 2:**
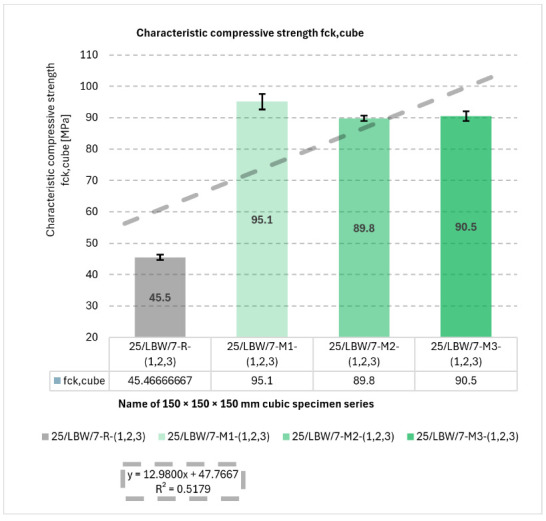
Results of mechanical strength tests under static external loading. Characteristic compressive strength (f_ck,cube_) of type “B” cubic specimens for 28-day cured concrete series R, M1, M2, and M3 [own analysis].

**Figure 3:**
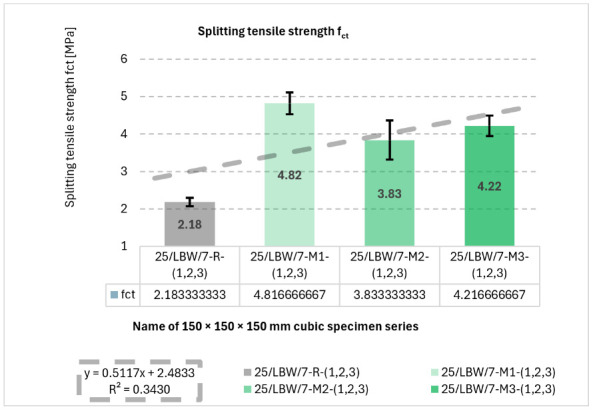
Results of mechanical strength tests under static external loading. Splitting tensile strength (f_ct_) of type “B” cubic specimens for 28-day cured concrete series R, M1, M2, and M3 [own analysis].

**Figure 4:**
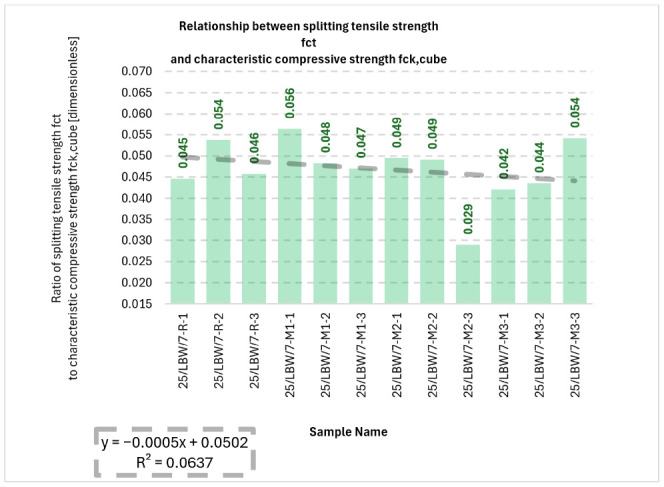
Bar chart illustrating the relationship between splitting tensile strength (f_ct_) and characteristic compressive strength (f_ck,cube_) for type “B” cubic specimens for 28-day cured concrete series R, M1, M2, and M3 [own analysis].

**Figure 5:**
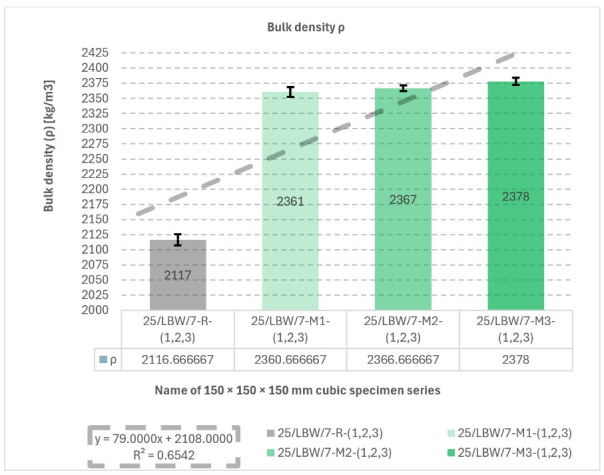
Results of bulk density (ρ) measurements. Bulk density (ρ) of type “B” cubic specimens for 28-day cured concrete series R, M1, M2, and M3 [own analysis].

**Figure 6:**
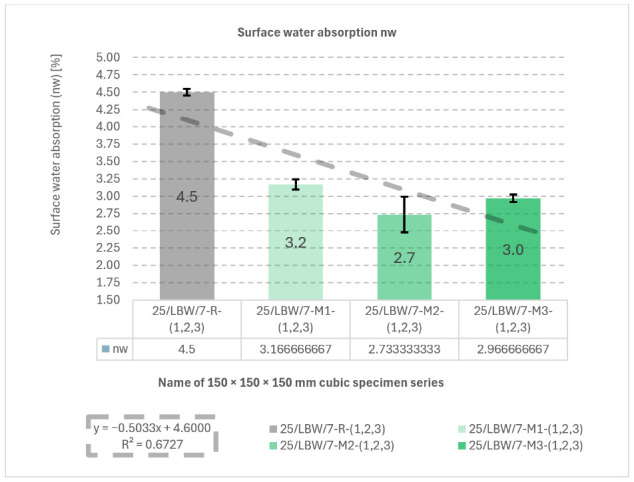
Results of surface water absorption (n_w_) tests. Surface water absorption (n_w_) of type “B” cubic specimens for 28-day cured concrete series R, M1, M2, and M3 [own analysis].

**Figure 7:**
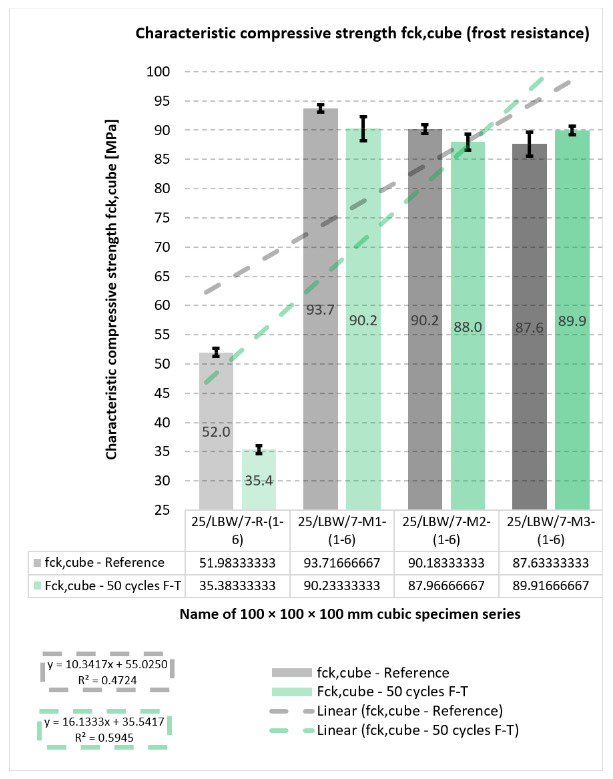
Results of tests on the reduction in characteristic compressive strength (f_ck,cube_) (internal frost resistance test of concrete after 50 freeze–thaw cycles) for type “C” cubic specimens from 56-day cured concrete series R, M1, M2, and M3 [own analysis].

**Figure 8:**
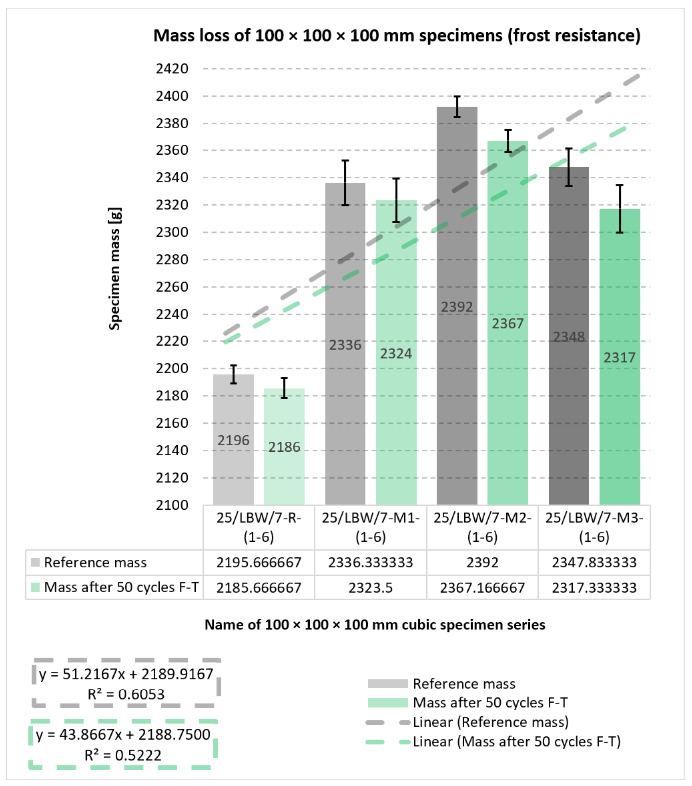
Results of mass change measurements (internal frost resistance test of concrete after 50 freeze–thaw cycles) for type “C” cubic specimens from 56-day cured concrete series R, M1, M2, and M3 [own analysis].

**Figure 9:**
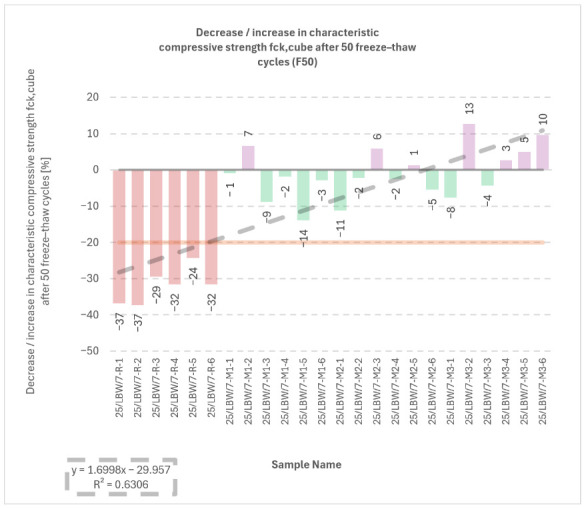
Results of tests on the reduction in characteristic compressive strength (f_ck,cube_) (internal frost resistance test of concrete after 50 freeze–thaw cycles) for type “C” cubic specimens from 56-day cured concrete series R, M1, M2, and M3 [own analysis].

**Figure 10:**
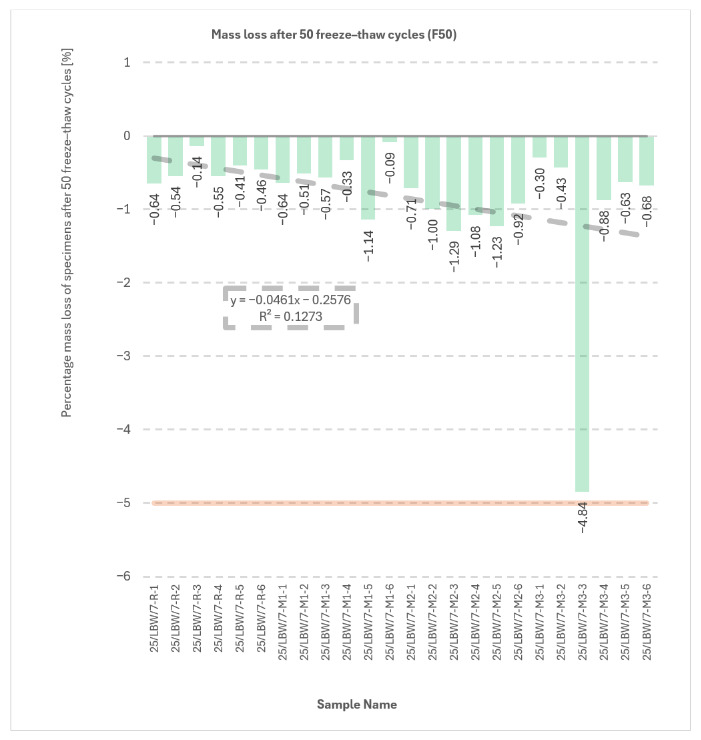
Results of mass loss measurements (frost resistance testing) of concrete after 50 freeze–thaw cycles) for type “C” cubic specimens from 56-day cured concrete series R, M1, M2, and M3 [own analysis].

**Figure 11:**
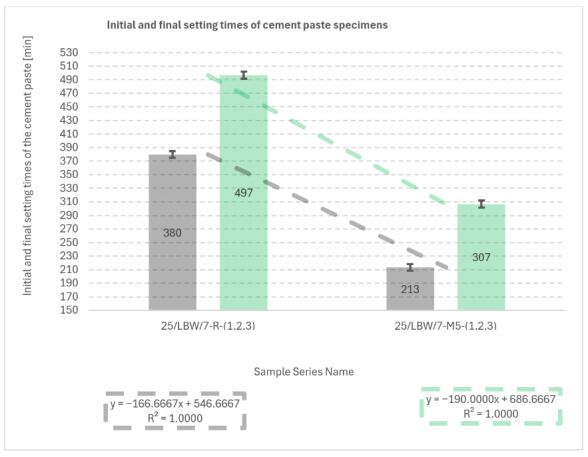
Results of tests determining the initial and final setting times of cement paste for truncated-cone specimens from 28-day cured concrete the reference and material-modified series (R and M5) [own analysis].

The curing period depended on the type of test performed. Compressive strength and splitting tensile strength tests were carried out on concrete specimens after 28 days of curing. The freeze–thaw resistance tests were performed after 56 days of curing, in accordance with the adopted durability-testing procedure. The notation used in Figure 1, Figure 2, Figure 3, Figure 4, Figure 5, Figure 6, Figure 7, Figure 8, Figure 9, Figure 10 and Figure 11 corresponds to the internal laboratory identification of the specimen series and does not directly denote the curing age.

The number of tested specimens also depended on the type of experiment. For the mechanical strength tests and the basic physical tests (Figure 2, Figure 3, Figure 4, Figure 5, Figure 6 and Figure 11), three specimens were tested for each concrete series, and the reported values correspond to the arithmetic mean with standard error bars. For the freeze–thaw durability assessment (Figure 7, Figure 8, Figure 9 and Figure 10), six specimens were used for each series. In the specimen labelling system, the notation (1,2,3) denotes the individual specimen numbers within a three-specimen test series, whereas (1–6) denotes the individual specimen numbers within a six-specimen test series used in freeze–thaw testing.

The adopted specimen types and testing programme were selected to ensure consistency with the intended performance assessment of the developed rapid-setting concrete and with the requirements of the applicable test standards. The geometry, preparation, curing, and testing procedures for fresh concrete, compressive strength, splitting tensile strength, and cement setting time were based on the relevant EN/PN-EN standard procedures, including EN 12350-2, EN 12350-7, EN 12390-1, EN 12390-2, EN 12390-3, EN 12390-4, EN 12390-6, and EN 196-3. The freeze–thaw resistance assessment was carried out according to the national procedure specified in PN-B-06265:2022-08, Annex N, in relation to the exposure and durability framework of EN 206+A2:2021 [38,39,40,41,42,43,44,45,46,47,48,49,50,51].

At the same time, the composition of the specimen series was not taken directly from a single standard but was developed as an original research programme based on the study objective. In particular, the mixture formulations were designed to evaluate the effect of a hybrid glass–granite aggregate skeleton, a CEM V multicomponent binder, and waste-derived sodium silicate/glass powder additives on the fresh-state behaviour, early setting, mechanical performance, durability, and microstructure of the resulting concrete. This design rationale was informed by previous literature on recycled-glass concrete, hydration acceleration, and particle-packing optimisation.

### 4.1. Concrete Mix Design Methodology—Synthetic Description

A multicriteria comparative assessment of the principal methods used for proportioning ready-mixed cement concrete was undertaken. On this basis, the B. Bukowski method was selected as the primary framework for the quantitative design of concrete mixtures based on hydraulic binders, including medium-strength ready-mixed concretes and vibro-compacted concrete mixtures.

The concrete mixtures were designed in accordance with the following fundamental assumptions:

- Concrete strength class: normal-weight concrete C30/37 (f_ck,cyl_ = 30 MPa; f_ck,cube_ = 37 MPa) with a bulk density ρ < 2600 kg/m^3^. Compliance with the target strength class was verified by mechanical testing after 56 days of curing using standard type “B” cubic specimens with actual dimensions of 150 × 150 × 150 mm (declared specimen dimensions);
- Consistency class: S1/S2, achieved through the application of a liquid chemical admixture exhibiting a strong water-reducing effect relative to the total reference mixing water content;
- Aggregate skeleton design: hybrid aggregate systems composed of crushed waste and mineral aggregates, specifically glass–granite aggregate skeletons;
- Use of waste-derived constituents: incorporation of recycled materials in the composition of the hybrid aggregate skeletons;
- Maximum nominal size of coarse aggregate: D_max_ = 8.0 mm (crushed granite);
- Environmental exposure conditions; consideration of combined exposure classes, including: XC3—concrete exposed to water action (increased air humidity), XD2—concrete exposed to chlorides other than from seawater, XF3—concrete exposed to severe freeze–thaw action (rainfall and freezing water); and XA2—chemical attack from soil and groundwater characterised by SO_4_^2−^ > 600 mg/L; SO_4_^2−^ ≤ 3000 mg/L; NH^4+^ > 30 mg/L; NH^4+^ ≤ 60 mg/L; Mg^2+^ > 1000 mg/L; Mg^2+^ ≤ 3000 mg/L; pH < 5.5; pH ≥ 4.5);
- Additional exposure class: XM2—associated with a high risk of surface abrasion, typical of pavements subjected to traffic loads from elastomeric tyres or steel rollers;
- Adoption of standard limiting values based on the combination of exposure classes: water-to-cement ratio w/c ≤ 0.50; minimum concrete strength class C30/37; minimum eco-cement content c = 320 kg/m^3^; air content in fresh concrete pm = 3.8–4.5% and in hardened concrete pb = 4.5–5.5%;
- Reduction in the reference total mixing water content through the use of a highly effective liquid chemical admixture improving concrete workability (superplasticiser based on an aqueous solution of modified polycarboxylate ethers);
- Chloride ion content classified as Cl 0.40, i.e., the maximum chloride ion content Cl^−^ relative to the reference cement mass equal to 0.40% (maximum total contribution of chloride ions from all mixture constituents).

The preliminary design stage involved the qualitative selection of constituents for hybrid glass–granite aggregate skeletons with constant bulk density (ρ = 2.65 kg/m^3^ for both granite aggregate 2.0/8.0 mm and soda–lime glass aggregate 0/2.0 mm). Percentage values of characteristic grading points were adopted for the aggregate skeletons as follows: Pp (filler point): 3.2% (0/0.063 mm; 0.063/0.125 mm), PpP (filler–sand point): 19.7% (0/0.063 mm; 0.063/0.125 mm; 0.125/0.250 mm; 0.250/0.500 mm), PP (sand point): 50.0% (0/0.063 mm; 0.063/0.125 mm; 0.125/0.250 mm; 0.250/0.500 mm; 0.500/1.0 mm; 1.0/2.0 mm). Granulometric analyses were conducted on representative samples of crushed soda–lime glass aggregate (0/2.0 mm) and mineral coarse aggregate in the form of granite (2.0/8.0 mm). The resulting hybrid aggregate skeletons were further refined by the addition of waste filler (0/0.063 mm) and soda–lime glass powder (0/0.250 mm). Standard limiting grading curves for the 0/8.0 mm fraction were applied, ensuring that the designed grading curve remained within the well-graded zone bounded by the lower- and middle limiting curves. An auxiliary coefficient, X1, defining the relationship between the sand point of granite (PP1) and that of the glass aggregate (PP2) relative to the sand point of the entire aggregate skeleton (PP), was calculated. On this basis, appropriate proportions of fine and coarse aggregates within the 0/8.0 mm skeleton were established.

The water demand of the aggregate skeleton and dry constituent mixture was determined using flow indices derived from O. Stern’s tables. In addition, empirical relationships were applied to accurately estimate the water demand of the eco-cement (w_cem_), soda–lime glass filler (w_ps_), and soda–lime glass powder (w_ms_). Water demand indices were selected with reference to the assumed S1 consistency class. An increase in mixture flowability was achieved by incorporating a liquid chemical admixture based on an aqueous solution of modified polycarboxylate ethers, resulting in S2 consistency according to the slump test (w/c = 0.42). Two correction factors were introduced into the final flow index calculations: c_1_—correction for the use of crushed aggregates, c_2_—correction accounting for differences in bulk density between crushed aggregates and reference natural aggregates. Using the equations of the B. Bukowski method, preliminary (pre-correction) mass proportions of the reference concrete mix were calculated for eco-cement CEM V/A (S-V) 42.5 N-LH/HSR/NA, granite aggregate 2.0/8.0 mm, soda–lime glass aggregate 0/2.0 mm, and total mixing water. Figure 1 illustrates the designed grading curve and the corresponding characteristic points (Pp, PpP and PP).

The designed concrete mix compositions are presented in Table 1. In this study, “glass dust” denotes the finest ground fraction of soda–lime waste glass with particle size 0.0/0.063 mm, whereas “glass powder” denotes the coarser ground fraction with particle size 0.0/0.250 mm (both obtained by grinding and sieving of waste soda–lime glass).

### 4.2. Characteristics of Concrete Mix Constituents

Concrete mixtures were designed using a low-emission multicomponent eco-hydraulic binder, i.e., CEM V/A (S-V) 42.5 N–LH/HSR/NA (slag–fly ash cement), characterised by reduced soluble alkali content, low heat evolution, and enhanced sulphate resistance. The binder exhibited a Blaine specific surface area of 4950 ± 150 cm^2^/g, initial/final setting times of 285 ± 10 min and 360 ± 10 min, and a 28-day compressive strength of 58 ± 2 MPa.

The aggregate skeleton consisted of crushed granite 2.0/8.0 mm (coarse aggregate) and waste soda–lime glass granulate 0.0/2.0 mm (fine aggregate). The granite aggregate fulfilled typical performance requirements (e.g., LA30, very low chloride content, low absorption, and freeze–thaw category F1). The glass granulate was classified as Gf85 and exhibited an alkaline reaction (pH ≈ 10).

Tap water (≈21 °C, pH 7) was used as mixing water and as a hydration activator. Two liquid admixtures were applied: a PCE-based high-range water-reducing admixture at 1.50% (by binder mass) and a copolymer-based viscosity-modifying admixture at 1.00% (by binder mass).

Soda–lime glass dust (0/0.063 mm) and glass powder (0/0.250 mm) were dispersed in an aqueous sodium silicate solution (water glass; MR = 1.6, pH ≈ 12) introduced at 2.50% and 5.00% (by binder mass). Both glass fractions were dosed at 1.00% each (by binder mass).

### 4.3. Test Methods for Cement Paste and Concrete Mixtures

Initial and final setting times of cement pastes were determined using the Vicat method according to [26]. A controlled deviation from [38] was introduced by preparing pastes consistent with the designed compositions: a reference paste (R) and modified pastes (M4, M5) incorporating sodium silicate solution and dispersed glass dust/powder. Paste compositions are reported in Table S1 in the Supplementary Materials.

Concrete mix consistency was determined by the slump test according to [24,25]. The total air content of fresh concrete was measured using the pressure method according to [27].

### 4.4. Methods for Determining the Mechanical Strength of Concrete

Compressive strength was determined on 150 × 150 × 150 mm cubes under axial loading using a calibrated testing machine, with a controlled stress rate, following the procedure described in [38,39,40,41] (including specimen geometry/positioning checks in [42,43,44,45]). Splitting tensile strength was determined using the Brazilian test, employing bearing strips and a positioning frame, according to [46,47,48]; values were calculated from the failure load.

### 4.5. Methods for Assessing Concrete Durability

Water absorption was determined on 150 × 150 × 150 mm cubes after 56 days of curing by oven-drying to constant mass and subsequent water saturation to mass stabilisation; calculations followed [49].

Freeze–thaw resistance was assessed for F50 exposure using 100 × 100 × 100 mm cubes after 56-day curing, quantifying mass change and residual compressive strength after 50 cycles of freezing and thawing; the procedure and conformity assessment followed [50,51].

### 4.6. Methodology of Microstructural Analysis

Microstructural analysis was carried out on hardened concrete specimens after curing using a Keyence VHX-7200 digital microscope operated in reflected-light mode. Representative surfaces of the reference concrete and selected modified concretes were examined in order to assess the morphology of the cement matrix, the aggregate–paste interfacial zones, the occurrence of microcracks, and the size and distribution of macroscopic air voids. The observations were performed at optical magnifications ranging from 20× to 500×. In addition to conventional 2D imaging, selected observations included 3D surface topography and surface roughness visualisation, which supported the qualitative interpretation of matrix densification and interfacial integrity.

## 5. Results

Experimental results were analysed using descriptive statistics (mean values reported with standard error bars) and linear regression with the coefficient of determination (R^2^). Statistical inference and uncertainty estimation were performed at a 95% confidence level using a Type A approach (coverage factor *k* for *p* = 0.95). Summary datasets are provided in the tables referenced below.

### 5.1. Characteristic Compressive Strength (f_ck,cube_) of Concrete Specimens

Results for f_ck,cube_ are shown in Figure 2. Series M1 achieved the highest mean compressive strength (95.1 ± 2.4 MPa), whereas the reference series R showed the lowest value (45.5 ± 0.8 MPa), indicating an approximately 2.1-fold increase for M1 relative to R.

A linear trend was observed (*y* = 12.9800*x* + 47.7667; R^2^ = 0.5179), indicating increased f_ck,cube_ with aggregate-skeleton modification in the hybrid glass–granite system.

The positive linear trend obtained for f_ck,cube_ suggests that the applied modification of the hybrid glass–granite aggregate skeleton generally contributed to strength enhancement within the investigated series. However, the coefficient of determination (R^2^ = 0.5179) indicates only a moderate linear fit, which means that the strength development cannot be explained solely by a simple proportional increase in the modification level. This is consistent with the existence of an optimum composition, for which the beneficial packing and interfacial effects are maximised.

Individual values of mechanical strength measurements under static external loading and the results of the frequency analysis for the characteristic compressive strength (f_ck,cube_) of cubic concrete specimens are presented in Tables S2–S6, which can be found in the Supplementary Materials.

The final results of the characteristic compressive strength (f_ck,cube_) tests are summarised in the consolidated Table 2, which presents the descriptive statistical parameters of the measured data.

### 5.2. Splitting Tensile Strength (f_ct_) of Concrete Specimens—Brazilian Test

Splitting tensile strength results are presented in Figure 3. The highest mean f_ct_ was obtained for M1 (4.82 ± 0.29 MPa), while R exhibited the lowest value (2.18 ± 0.11 MPa). The fitted linear trend (*y* = 0.5117*x* + 2.4833) yielded R^2^ = 0.3430.

For splitting tensile strength, the positive slope of the regression line indicates that the material modifications generally improved the tensile response of the concrete. Nevertheless, the relatively low coefficient of determination (R^2^ = 0.3430) suggests that this relationship is weaker than in the case of compressive strength, which may reflect the greater sensitivity of tensile performance to local defects, interfacial discontinuities, and specimen-scale heterogeneity.

The relationship between f_ct_ and f_ck,cube_ is summarised in Figure 4. The f_ct_/f_ck,cube_ ratio ranged from 0.029 to 0.056 (2.90–5.60%). The ratio showed a slight decreasing trend (*y* = −0.0005*x* + 0.0502) with low explanatory power (R^2^ = 0.0637), indicating relative stability of the ratio across series.

The very low value of the coefficient of determination for the f_ct_/f_ck,cube_ ratio (R^2^ = 0.0637) indicates that no meaningful linear dependence was identified between this ratio and the investigated series arrangement. In practical terms, this means that although both compressive and tensile strengths changed between mixtures, their mutual proportion remained relatively stable across the tested concretes.

Individual test results for the splitting tensile strength (f_ct_) are presented in Table S9, which can be found in the Supplementary Materials.

Four frequency tables (Tables S10–S13) were prepared to present the class intervals of the measured splitting tensile strength (f_ct_) values. Tables S10–S13 are in the Supplementary Materials.

Descriptive statistics for the splitting tensile strength (f_ct_) were developed and are presented in Table S14 in the Supplementary Materials.

### 5.3. Bulk Density (ρ) of Concrete Specimens

Bulk density results are shown in Figure 5. The highest mean density was recorded for M3 (2378 ± 6 kg/m^3^), whereas the reference series R exhibited the lowest value (2117 ± 9 kg/m^3^). The linear fit (*y* = 79.0000*x* + 2180.0000) yielded R^2^ = 0.6542, indicating an increasing trend in ρ across the investigated series.

The increasing linear trend observed for bulk density indicates that the modified mixtures tended to become more compact as the aggregate system was altered. The corresponding coefficient (R^2^ = 0.6542) points to a moderately strong relationship, suggesting that the hybrid aggregate modification had a relatively consistent effect on the packing density of the concrete.

Individual bulk density (ρ) measurement results and descriptive statistics for bulk density (ρ) were developed are presented in Tables S15 and S16 in the Supplementary Materials.

### 5.4. Surface Water Absorption (n_w_) of Concrete Specimens

Surface water absorption results are presented in Figure 6. The reference series R showed the highest mean n_w_ (4.50 ± 0.05%), whereas M2 achieved the lowest value (2.73 ± 0.26%). The fitted trend line (*y* = −0.5033*x* + 4.6000) yielded R^2^ = 0.6727, indicating a decreasing trend in n*_w_* for modified mixtures.

The negative linear trend obtained for surface water absorption shows that the material modifications were generally associated with reduced water uptake. Since the regression fit is relatively pronounced (R^2^ = 0.6727), this result supports the interpretation that the modified aggregate skeleton improved matrix compactness and reduced the effective accessibility of pores to water ingress. This trend is also consistent with the observed increase in density and strength for the modified series.

Individual surface water absorption (n_w_) measurement results and descriptive statistics for surface water absorption (n_w_) were developed are presented in Tables S17 and S18.

### 5.5. External and Internal Frost Resistance of Concrete Specimens

Durability outcomes after freeze–thaw exposure are shown in Figure 7, Figure 8, Figure 9 and Figure 10. The internal frost resistance assessment (50 cycles) revealed a pronounced reduction in f_ck,cube_ for the reference series (35.4 ± 0.7 MPa), while modified series maintained substantially higher post-cycling strengths (M1: 90.2 ± 2.1 MPa; M2: 88.0 ± 1.4 MPa; M3: 89.9 MPa, as reported).

Mass change measurements after 50 cycles showed losses of approximately −10 g (R), −13 g (M1), −25 g (M2), and −31 g (M3), consistent with surface scaling.

With respect to the compressive-strength reduction criterion (limit 20%), the reference series R did not satisfy the requirement, whereas M1–M3 complied, with reductions reported in the range of approximately −1.0% to −14.0%. All series met the mass-loss criterion (≤5.0%); the maximum reported loss reached −4.84% for specimen No. 3 in series M3.

The relationships shown in Figure 7, Figure 8 and Figure 9 indicate that the modified concretes retained substantially better frost-related performance than the reference series after freeze–thaw exposure. In particular, the post-cycling residual compressive strength remained high for M1–M3, whereas the reference concrete exhibited a much stronger deterioration. At the same time, the mass-change results indicate that freeze–thaw resistance cannot be interpreted solely through a single monotonic parameter, because the modified concretes combined high residual strength with varying scaling-related mass losses. This confirms that the durability response is governed by a combination of matrix compactness, interfacial integrity, and surface damage sensitivity.

Descriptive statistics for the characteristic compressive strength (f_ck,cube_) of specimens from series R, M1, M2, and M3 (internal frost resistance testing of concrete prior to 50 F–T cycles) and Descriptive statistics for the characteristic compressive strength (f_ck,cube_) of specimens from series R, M1, M2, and M3 (internal frost resistance testing of concrete after 50 freeze–thaw cycles) are presented in Tables S19 and S20 in the Supplementary Materials.

Descriptive statistics for specimen mass values obtained for series R, M1, M2, and M3 (internal freeze–thaw resistance testing of concrete prior to 50 F–T cycles) and descriptive statistics for changes in specimen mass for series R, M1, M2 and M3 (internal freeze–thaw resistance test of concrete after 50 freeze–thaw cycles) are presented in Tables S21 and S22 in the Supplementary Materials.

### 5.6. Initial and Final Setting Times of Cement Paste Specimens

Initial and final setting times are summarised in Figure 11. Series M4 was excluded, as both setting times exceeded those of the reference paste (R), indicating excessive retardation and thus an unfavourable formulation.

Relative to the reference paste (R: 380 ± 5 min initial and 497 ± 5 min final), the modified paste M5 exhibited markedly shortened setting (213 ± 5 min initial and 307 ± 5 min final), corresponding to reductions of 167 min (initial) and 190 min (final). Standard errors prior to rounding were 3 min for all series; values are reported with rounding to 5 min.

Correlation analysis between setting-time variables was performed; however, due to the limited dataset (N = 3 per series) and the use of only two mix series in the final comparison (R and M5), the derived relationships were considered statistically non-representative and no causal inference was formulated.

Individual readings, correlation matrices/results and extended descriptive statistics are presented in Tables S23–S27 in the Supplementary Materials.

### 5.7. Correlation Between Glass Content and Key Performance Parameters

To better interpret the role of waste glass in the hybrid aggregate skeleton, a correlation analysis was performed between the volumetric glass content and selected performance parameters, including compressive strength and water absorption. The results show a clearly non-linear relationship between glass content and mechanical performance. An increase in glass content from 0 to 5 vol.% resulted in a pronounced strength gain, whereas further increases to 10–15 vol.% led to a gradual reduction in strength. This behaviour confirms the existence of an optimum glass dosage and supports the interpretation that moderate glass incorporation improves packing density, while excessive replacement negatively affects interfacial performance.

A similar trend was observed for water absorption. Mixtures with low glass content exhibited reduced absorption compared with the reference concrete, suggesting a denser microstructure and lower effective porosity. At higher glass contents, the absorption tendency stabilised or slightly increased, which may be associated with local heterogeneity of the aggregate skeleton and microstructural discontinuities near glass particles. The combined correlation analysis indicates that the best balance between strength and transport-related durability indicators is achieved at approximately 5 vol.% glass aggregate.

### 5.8. Microstructural Analysis of Concrete

**Figure 12:**
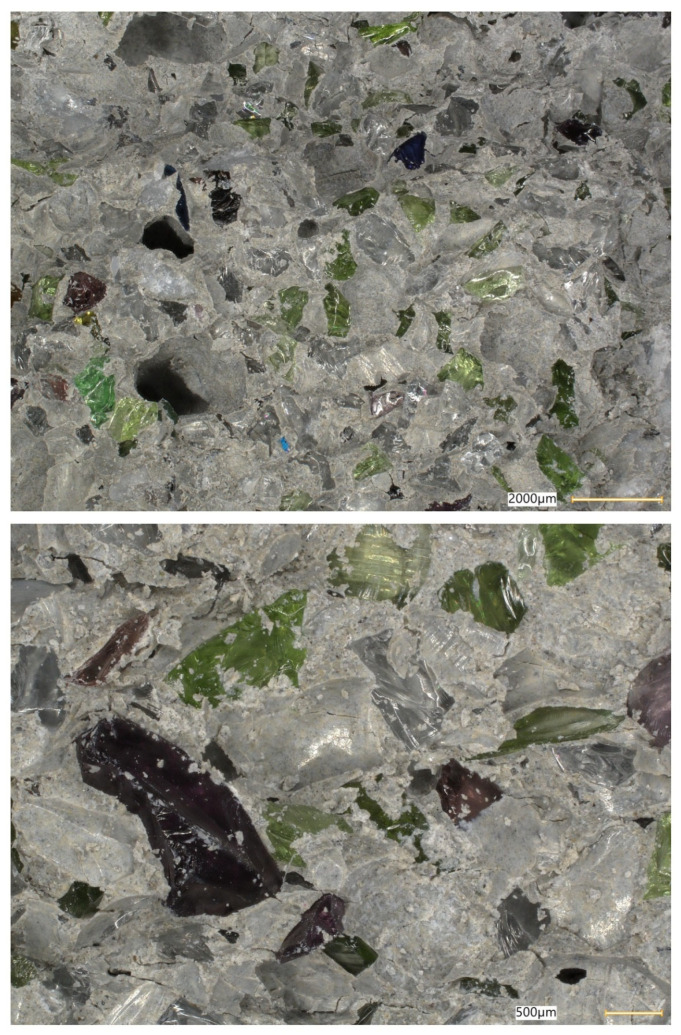
Microscopic observation using a Keyence VHX-7200 digital microscope. Representative images of the macrostructure of the reference concrete (R). Soda–lime glass particles embedded in the cement matrix; material imperfections in the form of negative macroscopic air voids; microcracks in the cement matrix (reflected-light images, optical magnifications 20× and 50×) [own analysis].

**Figure 13:**
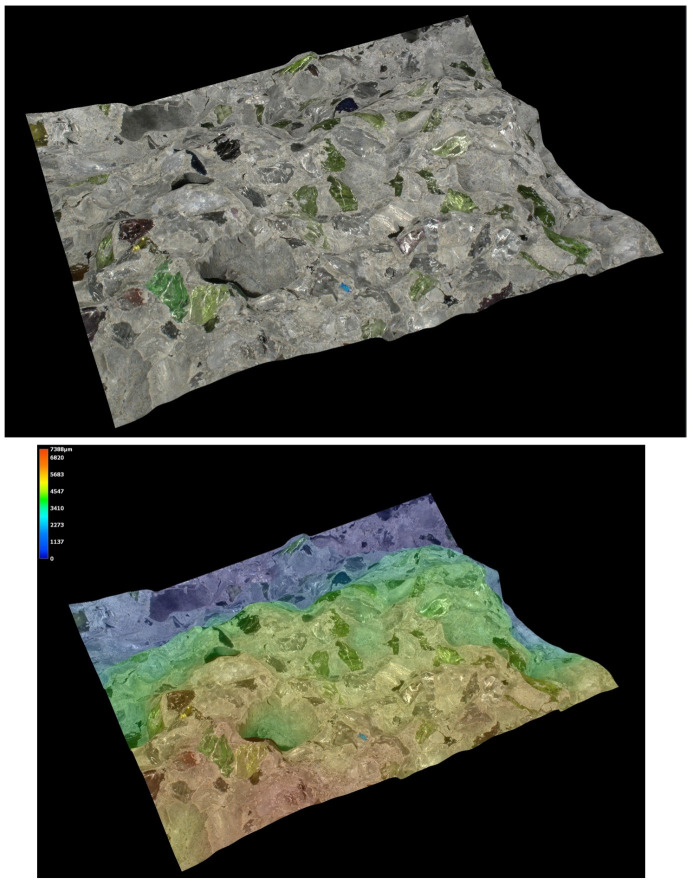
Microscopic observation using a Keyence VHX-7200 digital microscope. Representative images of the macrostructure of the reference concrete (R). 3D surface topography model of the cement matrix and crushed glass and granite aggregates. Hypsometric (height-map) visualisation of the matrix surface topography using a “Rainbow” scale (reflected-light images, optical magnifications 20× and 50×) [own analysis].

**Figure 14:**
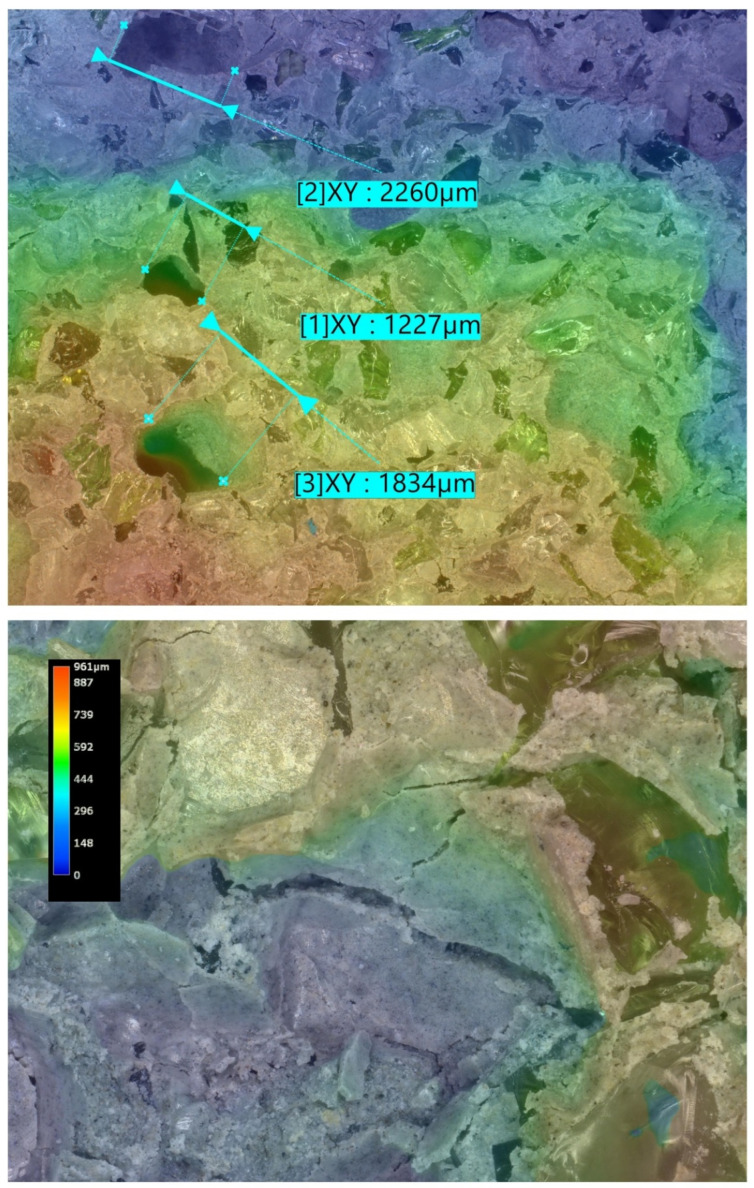
Microscopic observation using a Keyence VHX-7200 digital microscope. Representative images of the macrostructure of the reference concrete (R). Surface topography model of the cement matrix and crushed glass and granite aggregates. Hypsometric (height-map) visualisation of the matrix surface topography using a “Rainbow” scale. Linear-dimension measurements of macroscopic air voids (reflected-light images, optical magnifications 20× and 100×) [own analysis].

**Figure 15:**
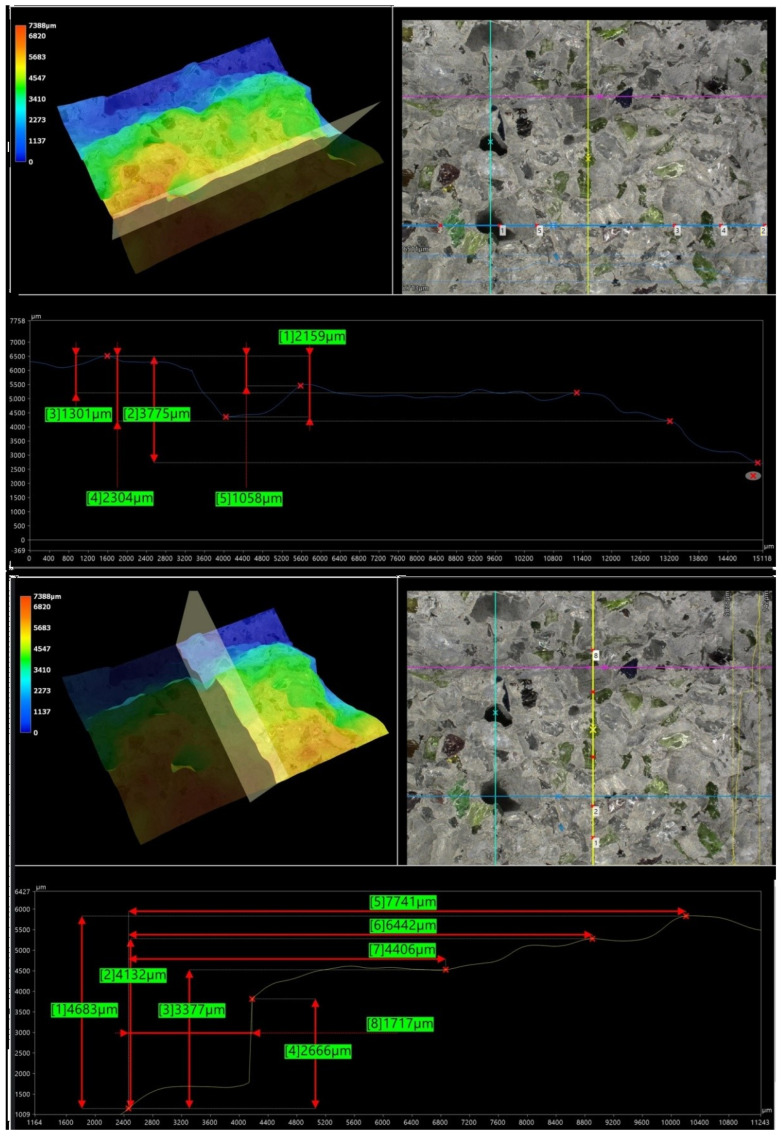
Microscopic observation using a Keyence VHX-7200 digital microscope. Representative images of the macrostructure of the reference concrete (R). Surface topography model of the cement matrix and crushed glass and granite aggregates. Hypsometric (height-map) visualisation of the matrix surface topography using a “Rainbow” scale. Linear-dimension measurements on the cement matrix surface (reflected-light images, optical magnification 20×) [own analysis].

**Figure 16:**
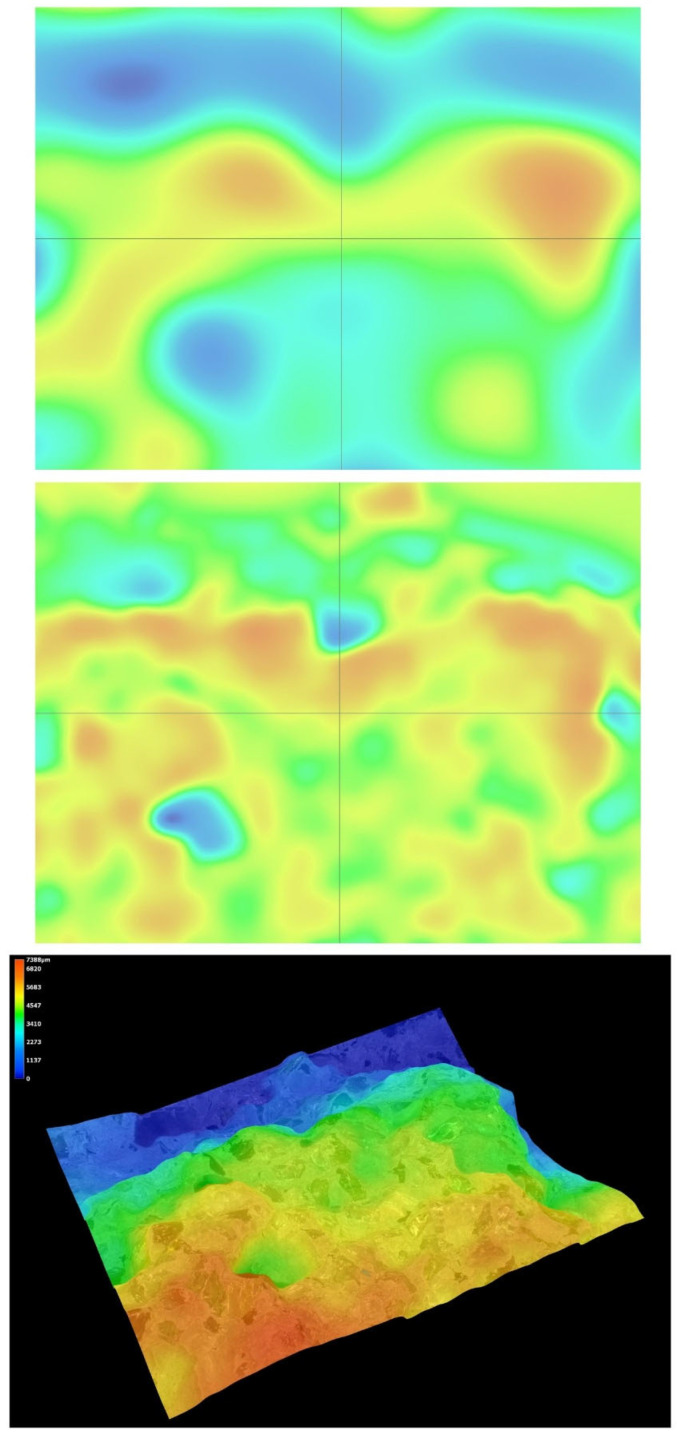
Microscopic observation using a Keyence VHX-7200 digital microscope. Representative images of the macrostructure of the reference concrete (R). Surface roughness model of the cement matrix and crushed glass and granite aggregates. Hypsometric (height-map) visualisation of the matrix surface topography using a “Rainbow” scale (reflected-light images, optical magnification 20×) [own analysis].

**Figure 17:**
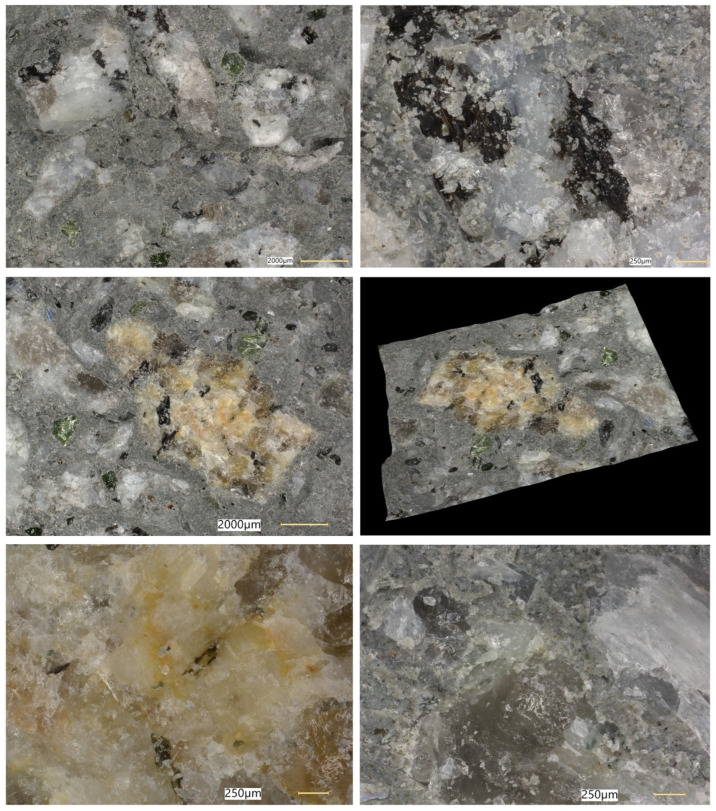
Microscopic observation using a Keyence VHX-7200 digital microscope. Representative images of the macrostructure of the waste-component-modified concrete (M1). Imaging of the cement matrix surface, granite aggregate, and soda–lime glass granulate (reflected-light images, optical magnifications 20×–100×) [own analysis].

**Figure 18:**
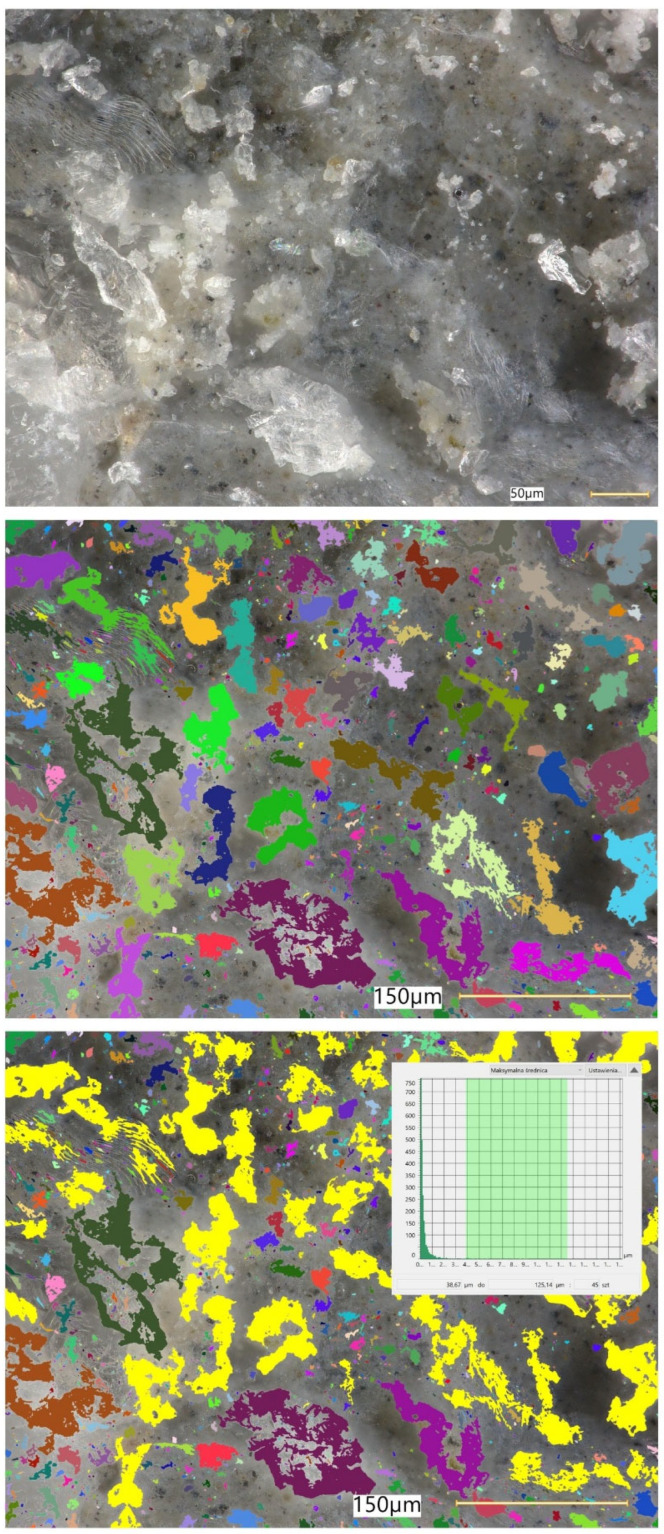
Microscopic observation using a Keyence VHX-7200 digital microscope. Representative images of the macrostructure of the waste-component-modified concrete (M1). Imaging of the cement matrix surface, granite aggregate, and soda–lime glass granulate (reflected-light images, optical magnification 500×) [own analysis].

**Figure 19:**
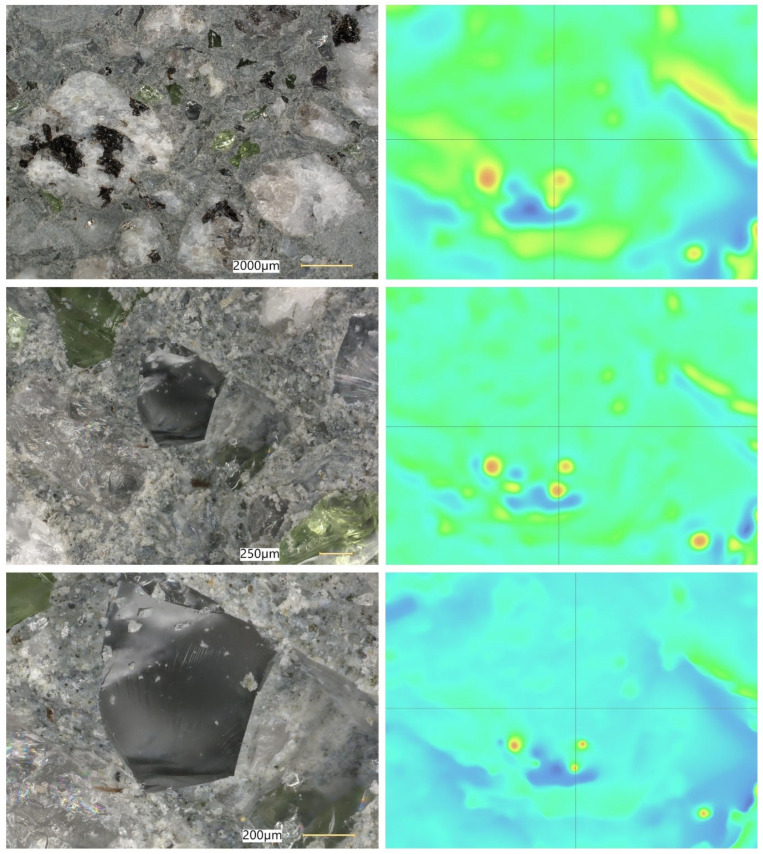
Microscopic observation using a Keyence VHX-7200 digital microscope. Representative images of the macrostructure of the waste-component-modified concrete (M2). Imaging of the cement matrix surface, granite aggregate, and soda–lime glass granulate (reflected-light images, optical magnifications 20×–200×) [own analysis].

**Figure 20:**
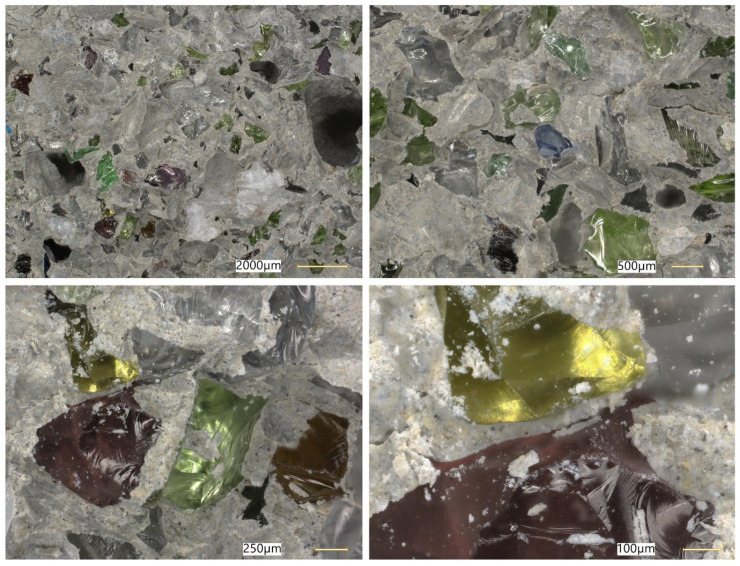
Microscopic observation using a Keyence VHX-7200 digital microscope. Representative images of the macrostructure of the waste-component-modified concrete (M3). Imaging of the cement matrix surface, granite aggregate, and soda–lime glass granulate (reflected-light images, optical magnifications 20×–300×) [own analysis].

Microstructural observations were performed on hardened specimens of series R and M1–M3 using a Keyence VHX-7200 digital microscope in reflected-light mode (magnification range 20×–500×). The analysis focused on qualitative assessment of the cement matrix morphology, aggregate–paste interfacial zones, microcracks, macroscopic air voids, and selected 3D topography, roughness visualisations to support comparative interpretation of compactness and interfacial integrity (Figure 12, Figure 13, Figure 14, Figure 15, Figure 16, Figure 17, Figure 18, Figure 19 and Figure 20).

Figure 16 provides an additional roughness-based representation of the reference concrete (R) surface and complements the observations presented in Figure 12, Figure 13, Figure 14 and Figure 15. The hypsometric roughness map indicates a pronounced topographic heterogeneity of the cement-matrix surface and the neighbouring aggregate zones, which is consistent with the presence of local imperfections, air voids, and microcracking identified in the preceding images. In qualitative terms, this roughness pattern suggests a less compact and less uniform microstructure of the reference concrete, which helps explain its lower mechanical performance and less favourable transport-related behaviour compared with the modified mixtures.

Figure 12, Figure 13, Figure 14, Figure 15 and Figure 16 present the microstructural characteristics of the reference concrete (R). The observed images reveal the presence of macroscopic air voids, local matrix imperfections, and microcracks within the cement matrix. The 3D topography and surface roughness visualisations additionally indicate a relatively heterogeneous surface morphology, which may reflect lower matrix compactness and weaker interfacial integrity.

In contrast, the modified concrete M1 shown in Figure 17 and Figure 18 exhibits a more compact and homogeneous microstructural arrangement. The cement matrix appears denser, and the contact zones between the matrix and the aggregate particles are visually more continuous. This observation is consistent with the mechanical test results, in which M1 achieved the highest compressive and splitting tensile strength, and with the reduced water absorption reported for the low-glass-content mixture.

The observations for M2 and M3 (Figure 19 and Figure 20) suggest that increasing the glass aggregate content above the optimum level leads to a less favourable microstructural arrangement. Although the matrix remains generally compact, a higher proportion of glass particles may contribute to local heterogeneity of the aggregate skeleton and to the formation of discontinuities in the aggregate–paste transition zones. This interpretation is consistent with the gradual reduction in strength observed for M2 and M3 compared with M1, despite their still satisfactory absolute performance. Overall, the microstructural observations support the conclusion that a moderate glass aggregate replacement level improves packing density and matrix integrity, whereas excessive replacement may reduce the beneficial effect.

## 6. Discussion

The obtained results confirm that a properly designed hybrid aggregate skeleton and the use of waste glass can significantly modify the mechanical properties of concrete. Particularly favourable effects were observed for mixture M1, in which 5% of the volume of natural aggregate was replaced with soda–lime glass granulate. The mean compressive strength f_ck,cube_ obtained for this series was more than twice that of the reference mixture, indicating very good packing of the granular skeleton and a potentially beneficial influence of the glass on the microstructure of the cement paste.

Further increasing the proportion of glass aggregate (M2, M3) led to a gradual reduction in compressive strength; however, the absolute strength values remained high relative to class C30/37. This suggests the existence of an optimal range of glass content, beyond which adverse effects begin to dominate, such as increased susceptibility to microcracking in the interfacial transition zone or deterioration of the mixture compaction conditions. This observation is consistent with reports in the literature, which indicate the need to limit the glass content to the order of several tens of percent by mass or volume of aggregate in order to avoid an excessive reduction in mechanical properties and the risk of alkali–silica reaction (ASR).

The analysis of the splitting tensile strength (f_ct_) results confirmed an overall increasing trend relative to the reference mixture, with the f_ct_/f_ck,cube_ ratio ranging from approximately 2.9 to 5.6%. These values are close to those typically reported for high-performance concretes, which may indicate a favourable internal structure and adequate bond between the cement paste and the glass aggregate. The applied linear models and the corresponding coefficients of determination (R^2^) demonstrate clear strength-increase trends as a function of mixture modification, while maintaining an acceptable level of result variability.

An important aspect from the perspective of practical applications is the effect of silicate-based additives (M4, M5) on the setting time and durability of concrete. The sodium silicate solution combined with glass powder and glass flour played a dual role: acting both as a setting accelerator and as a mineral additive contributing to microstructural densification. The reduction in setting time and the accelerated development of early-age strength are particularly desirable under post-disaster conditions, where rapid restoration of the load-bearing capacity of structural elements is required. At the same time, it is essential to maintain a balance between the rate of hydration and the associated heat release in order to minimise the risk of thermal cracking and long-term structural degradation.

The durability test results, including water absorption, frost resistance, and the assessment of alkali–silica reactivity (ASR), indicate that with an appropriate selection of glass fractions and binder types, it is possible to maintain performance parameters in compliance with the requirements of exposure classes XC3/XD2/XF3/XA2. This is particularly important in the context of the intended application of these mixtures in environments subjected to cyclic freeze–thaw action and exposure to aggressive ground- and groundwater solutions.

Building on the above application context, the presented results highlight a clear optimum content of waste glass within the aggregate skeleton. The maximum strength gain observed for M1 (5 vol.% glass aggregate) suggests that, at low replacement levels, the hybrid glass–granite skeleton benefits from improved grading and packing, while the detrimental mechanisms associated with higher glass contents remain limited. From the perspective of granular mechanics, the effect can be interpreted as a shift toward a denser particle arrangement and a lower volume of capillary-accessible pores, which increases the effective load-bearing area and reduces stress concentrations within the hardened composite. At the same time, limited glass incorporation can act as a “fine-tuning” component in the skeleton, filling voids between angular granite particles without disrupting the force chains and inter-particle interlock that typically govern the compressive performance of conventional concretes.

This interpretation is consistent with the observed trend of decreasing strength for mixtures M2 and M3 (10–15 vol.%). At higher glass contents, several effects may become dominant. First, the surface morphology and stiffness contrast between soda–lime glass and crushed granite can unfavourably influence the interfacial transition zone (ITZ). Smooth glass surfaces provide lower mechanical interlock and can promote localised debonding under load, especially when combined with a less favourable packing state. Second, the higher proportion of glass particles increases the probability of microcrack initiation at the paste–glass interface and subsequent crack propagation along weaker interfacial paths, which is particularly relevant under splitting tensile loading where the crack plane often traverses ITZ regions. Third, higher glass contents may reduce the overall frictional resistance of the aggregate skeleton and modify compaction behaviour, resulting in locally heterogeneous density. In practical terms, the results indicate that glass incorporation should be treated as a design variable with a non-linear response, rather than a simple replacement strategy: beyond a certain threshold, the balance between packing benefits and interfacial weakening shifts toward the latter. This has direct implications for field-oriented mix design, where the target should be an “effective” glass dosage that optimises early strength while avoiding excessive sensitivity to compaction quality and curing variability.

From the durability perspective, the favourable performance of the modified mixtures can be associated with the combined effect of the low-emission multicomponent CEM V binder system and the adopted hybrid aggregate skeleton concept. The reported compliance with the requirements of exposure classes XC3/XD2/XF3/XA2 is particularly relevant for rapid-repair materials that must withstand cyclic freeze–thaw action, chloride-bearing environments, and aggressive groundwater conditions. In such scenarios, durability is governed not only by the intrinsic chemical resistance of binder phases, but also by the transport properties of the composite (permeability, absorptivity, connectivity of capillary pores) and the stability of the pore system under cyclic saturation. The observed relationship between mechanical performance and durability indicators suggests that mixtures achieving high early strength do so in part through a denser microstructure, which can also reduce water ingress and limit the degree of saturation—both beneficial for freeze–thaw resistance. However, these benefits must be interpreted together with early-age behaviour: in time-critical repairs, high early compressive strength supports the accelerated reopening of infrastructure elements, but it must be balanced against the risk of premature cracking under restrained conditions, temperature gradients, and variable saturation. Therefore, field deployment should integrate structural requirements (early strength gain) with construction logistics (placement/finishing time) and exposure risk (moisture and freeze–thaw severity).

The setting-time results underline that the sodium silicate-based system requires a strict dosage window. In the cement paste series, the lower dosage (M4) led to an excessive prolongation of setting and was therefore rejected, while the higher dosage (M5) significantly shortened both initial and final setting times compared with the reference paste. This behaviour indicates that the interaction between soluble silicates and a multicomponent binder is not monotonic and may involve competing effects. At lower dosages, sodium silicate can modify the ionic environment and dispersion state of cement particles, potentially delaying the formation of a percolated hydration product network responsible for stiffening. At higher dosages, the increased availability of soluble silicate species may promote faster precipitation of C–S–H-type phases and accelerate the transition from a plastic to a rigid structure, especially in systems where additional reactive surfaces are present. Importantly, accelerated setting is not automatically synonymous with better performance: a very short setting time can reduce workable time, impair compaction, and increase the risk of cold joints or inadequate finishing. Moreover, rapid stiffening may increase susceptibility to early-age shrinkage and cracking if curing and thermal control are insufficient. Consequently, mixture selection for field deployment should not rely solely on achieving the shortest setting time, but should also consider workability retention, temperature sensitivity, curing feasibility, finishing time, and the cracking risk under realistic restraint conditions.

Microstructural observations reported in this work indicate overall densification and a reduced proportion of unfavourable air voids exceeding 300 μm, which is consistent with the improved tensile-to-compressive strength ratio and the enhanced freeze–thaw performance of the modified series. The microstructural argument becomes particularly important in view of the optimum behaviour of M1: if the paste matrix is denser and the ITZ remains sufficiently compact at low glass contents, strength and freeze–thaw resistance can improve simultaneously; if, however, ITZ defects and microcracks become more frequent at higher glass contents, strength decreases even if the matrix itself remains relatively dense. To strengthen the structure–property linkage and directly address reviewer expectations regarding SEM/EDS evidence, the discussion should explicitly link representative micrographs to macroscopic results by comparing the matrix and ITZ in the reference mix versus M1 (and optionally versus M3 as a “higher glass content” contrast), highlighting whether microcracks preferentially occur adjacent to glass particles or within the paste, and using EDS spectra/maps to characterise the chemistry of hydration products near glass inclusions. In particular, EDS evidence showing Ca–Si-rich hydration products surrounding glass particles (and any Na-enrichment associated with the silicate admixture) would support the proposed interpretation that the silicate system contributes to early-age structural development and densification. Conversely, if EDS indicates distinct interfacial discontinuities or chemically different zones at higher glass contents, this would provide a mechanistic explanation for the observed decline in strength.

Overall, the combined findings support the feasibility of designing rapid-setting concretes with a reduced environmental footprint by coupling CEM V cement with a hybrid glass–granite skeleton and a carefully dosed sodium silicate system. The results also suggest that the best-performing compositions are those that simultaneously optimise aggregate packing, preserve ITZ integrity, and maintain a pore system compatible with freeze–thaw exposure under realistic saturation conditions. This integrated interpretation provides a practical framework for future optimisation, where the key objective is not maximal glass substitution per se, but a controlled glass content that achieves the desired early strength and durability while maintaining constructability and robustness in field conditions.

The correlation analysis between glass content and performance parameters confirms that the mechanical and durability behaviour of the investigated concretes follows a non-linear trend with a distinct optimum. This finding is consistent with the microstructural interpretation proposed earlier: limited glass incorporation improves packing and matrix densification, whereas excessive glass content increases the likelihood of interfacial defects. From a practical standpoint, the correlation results reinforce the recommendation that glass dosage should be optimised rather than maximised, especially in rapid-repair concretes where both strength and durability must be achieved simultaneously.

In summary, the proposed solution—a fast-setting concrete based on CEM V/A (S-V) 42.5 N-LH/HSR/NA cement, incorporating a hybrid glass–granite aggregate skeleton and silicate-based additives—can be regarded as a promising alternative to conventional concretes for applications requiring rapid post-disaster infrastructure reconstruction. However, the study is limited by the relatively small number of mix design variants and the lack of a comprehensive analysis of the long-term effects of ASR. Future research should therefore focus on extending the scope of investigation to include fatigue testing, long-term exposure to corrosive environments, and numerical modelling of concrete behaviour under variable loading and temperature conditions.

From the sustainability perspective, the proposed concrete concept may offer advantages over conventional concrete made with high-clinker cement and exclusively natural aggregates. This potential benefit arises from the combined use of a multicomponent CEM V binder and waste-derived soda–lime glass as a partial aggregate substitute. According to available environmental product declaration data for cement produced in Poland, the average carbon footprint of CEM V is approximately 0.485 t CO_2_/t cement, compared with 0.710 t CO_2_/t cement for CEM I, indicating a substantially lower emission burden of the binder itself. In addition, the partial replacement of virgin aggregate by waste glass contributes to resource conservation and supports circular-material use. However, the overall environmental and economic performance of the developed concrete depends strongly on local conditions, including transport distances, waste-glass processing requirements, and the cost of sodium-silicate-based additives. Therefore, a full life-cycle assessment (LCA) and techno-economic analysis should be treated as a necessary next step of the research. From the economic perspective, the use of waste glass may reduce raw-material demand, although the final cost balance remains sensitive to local processing and logistics conditions.

The waste soda–lime glass considered in this study can typically be obtained from municipal and industrial recycling streams (e.g., container-glass cullet and flat-glass waste), provided that collection, sorting, and quality control are available. From a practical standpoint, the key constraints are the stability of supply and achievable fraction quality (contamination level, particle-size distribution, and moisture), as well as the need for crushing, milling and, where required, washing or additional separation. Consequently, the economic attractiveness of using waste glass in concrete is strongly region-dependent and is governed mainly by processing requirements and transport distances rather than by the waste material itself.

The obtained results should also be interpreted within the wider framework of contemporary research on eco-friendly construction materials containing recycled and waste-derived constituents. Beyond concrete technology itself, previous studies have reported the applicability of alternative building-material systems incorporating secondary raw materials, including paper-based composite materials and papercrete-type products [52,53]. Although these systems differ in composition and intended application from the rapid-setting concrete developed in the present study, they nevertheless confirm a broader engineering tendency toward waste valorisation, circular-material use, and reduction in the environmental burden associated with construction materials. Against this background, the proposed glass–granite–silicate concrete may be regarded as part of the ongoing transition toward more sustainable construction solutions.

## 7. Conclusions

Based on the laboratory investigations conducted, the following conclusions were drawn:

1. Mechanical properties

The use of hybrid glass–granite aggregate skeletons and waste sodium silicate resulted in a significant increase in the compressive strength and splitting tensile strength of concrete. For mixtures containing 5–15% (by volume) of glass granulate (M1–M3), the characteristic compressive strength f_ck,cube_ was up to approximately twice that of the reference mixture, while maintaining a favourable f_ct_/f_ck,cube_ ratio typical of high-performance concretes.

2. Durability

The incorporation of fine soda–lime glass granulate (0/2 mm) into the hybrid aggregate system contributed to a reduction in surface water absorption and an improvement in frost resistance. All modified mixtures (M1–M3) satisfied the requirements of the adopted exposure classes, and the mass loss due to scaling after 50 freeze–thaw cycles did not exceed the standard limit values.

3. Microstructure

The modified mixtures exhibited a reduced proportion of unfavourable air voids with diameters greater than 300 μm and an overall densification of the internal structure. No significant material defects were observed, such as agglomeration of glass powder and glass flour or excessive irregular macroporosity, confirming the validity of the adopted design concept for hybrid glass–granite aggregate skeletons.

4. Setting time and scope of application of the mixtures

The suspension of waste sodium silicate, used as a polydisperse medium for glass powder and glass flour, had a pronounced effect on the setting time of the cement paste. A dosage of 2.5% by mass of binder (M4) led to excessive prolongation of the setting time, whereas increasing the dosage to 5.0% (M5) resulted in a shortening of both the initial and final setting times, thereby limiting the universality of this mixture. Nevertheless, mixture M5 may be recommended for rapid repair and reconstruction of lightly reinforced or unreinforced concrete elements (e.g., walls, columns, stair flights, prefabricated pavement and drainage components), where rapid strength development is required and the degree of reinforcement is low.

5. Practical potential

The proposed eco-friendly rapid-setting concrete, based on CEM V/A (S-V) 42.5 N-LH/HSR/NA cement, a hybrid glass–granite aggregate skeleton, and waste silicate additives, represents a promising alternative to conventional concretes for applications involving emergency repair, rapid post-disaster reconstruction, and the restoration of infrastructure components exposed to adverse environmental conditions.

From a practical standpoint, the developed concrete should be understood as a material intended primarily for rapid emergency repair and local reconstruction works, rather than for universal application in all structural situations. Based on the obtained results, the most justified areas of use include lightly reinforced or unreinforced concrete elements, repair layers, small replacement members, stair flights, partition or retaining walls, pavement slabs, drainage components, and selected prefabricated elements used where rapid setting and early strength development are required. In contrast, the present results are not yet sufficient to recommend this material for heavily reinforced, massive, or critical primary structural members, for which additional structural, durability, and field-validation studies would be necessary.

6. Future research

Future development of the presented work is planned. The next stage of research should include broader long-term durability assessment, including extended freeze–thaw and moisture-related exposure scenarios; further optimisation of the glass–granite aggregate skeleton and waste-silicate additive content; validation of the proposed concrete under practical casting and application conditions and environmental and techno-economic assessment, including life-cycle and cost-related analysis of the developed material.

## Figures and Tables

**Table 1:** Mix compositions used for determining the initial and final setting times of cement pastes, including the reference mix (R) and mixes modified with waste-derived materials (M1–M5) [own analysis].

No.	Concrete Mix Component	Component Content in Concrete Mix [kg/m^3^]					
		R	M1	M2	M3	M4	M5
**1.**	Mixing water (tap water); pH = 7.0	169	169	169	169	169	169
**2.**	Hydraulic binder—special multicomponent cement containing slag and fly ash, type CEM V/A (S-V) 42,5 N-LH/HSR/NA	406	406	406	406	406	406
**3.**	Water-to-binder ratio, w/b [–]	0.42	0.42	0.42	0.42	0.42	0.42
**4** **.**	Liquid admixture No. 1—high-range water-reducing chemical admixture based on an aqueous solution of modified polycarboxylate ethers (dosage: 1.50% by mass of the cementitious binder)	6.10	6.10	6.10	6.10	6.10	6.10
**5** **.**	Liquid admixture No. 2—viscosity-modifying chemical admixture based on copolymers (dosage: 1.00% by mass of the cementitious binder)	4.06	4.06	4.06	4.06	4.06	4.06
**6** **.**	Aqueous sodium silicate solution (polydisperse medium for glass dust and glass powder; dispersing medium)	0	0	0	0	10.16	20.32
**7** **.**	Soda–lime glass **dust (0.0/0.063 mm)** (dispersed phase in an aqueous sodium silicate solution)	0	0	0	0	4.05	4.05
**8** **.**	Soda–lime glass **powder (0.0/0.250 mm)** (dispersed phase in an aqueous sodium silicate solution)	0	0	0	0	4.05	4.05
**9** **.**	Crushed fine aggregate—soda–lime glass aggregate, fraction 0.0/2.0 mm	873	90	178	268	873	873
**10** **.**	Crushed coarse aggregate—granite, fraction 2.0/8.0 mm	910	1693	1605	1515	910	910
**Total air-void content [%] per 1.0 m^3^ of concrete mixture/cement concrete**							
**11** **.**	Air content in fresh concrete	3.8	3.8	3.8	3.8	4.5	4.5
**12** **.**	Air content in concrete	4.5	4.5	4.5	4.5	5.5	5.5

**Table 2:** Descriptive statistics of measured characteristic compressive strength (f_ck,cube_) values for the R, M1, M2, and M3 specimen series [own analysis].

Descriptive Statistics					
Test Type: Characteristic Compressive Strength f_ck,cube_					
**Sample Series Name**	Number of Valid Samples (N)	Percentage of Valid Samples (%)	Arithmetic Mean	Confidence Limit (−95.0%)	Confidence Limit (95.0%)
25/LBW/7-R-(1,2,3)	3	100	45.5	41.2	49.7
25/LBW/7-M1-(1,2,3)	3	100	95.1	82.2	108.0
25/LBW/7-M2-(1,2,3)	3	100	89.8	85.4	94.2
25/LBW/7-M3-(1,2,3)	3	100	90.5	82.3	98.7
**Sample Series Name**	Trimmed Mean (5.0%)	Winsorized Mean (5.0%)	Geometric Mean	Harmonic Mean	Median
25/LBW/7-R-(1,2,3)	45.5	45.5	45.4	45.4	45.6
25/LBW/7-M1-(1,2,3)	95.1	95.1	95.0	94.9	96.7
25/LBW/7-M2-(1,2,3)	89.8	89.8	89.8	89.8	89.9
25/LBW/7-M3-(1,2,3)	90.5	90.5	90.5	90.4	89.5
**Sample Series Name**	Mode	Frequency of Mode	Minimum	Maximum	Lower Quartile
25/LBW/7-R-(1,2,3)	Multiple	1	43.7	47.1	43.7
25/LBW/7-M1-(1,2,3)	Multiple	1	89.3	99.3	89.3
25/LBW/7-M2-(1,2,3)	Multiple	1	88.0	91.5	88.0
25/LBW/7-M3-(1,2,3)	Multiple	1	87.8	94.2	87.8
**Sample Series Name**	Upper Quartile	10th Percentile	20th Percentile	30th Percentile	40th Percentile
25/LBW/7-R-(1,2,3)	47.1	43.7	43.7	43.7	45.6
25/LBW/7-M1-(1,2,3)	99.3	89.3	89.3	89.3	96.7
25/LBW/7-M2-(1,2,3)	91.5	88.0	88.0	88.0	89.9
25/LBW/7-M3-(1,2,3)	94.2	87.8	87.8	87.8	89.5
**Sample Series Name**	50th Percentile	60th Percentile	70th Percentile	80th Percentile	90th Percentile
25/LBW/7-R-(1,2,3)	45.6	45.6	47.1	47.1	47.1
25/LBW/7-M1-(1,2,3)	96.7	96.7	99.3	99.3	99.3
25/LBW/7-M2-(1,2,3)	89.9	89.9	91.5	91.5	91.5
25/LBW/7-M3-(1,2,3)	89.5	89.5	94.2	94.2	94.2
**Sample Series Name**	Range	Interquartile Range	Variance	Standard Deviation	Confidence Interval of the Standard Deviation (−95.0%)
25/LBW/7-R-(1,2,3)	3.4	3.4	2.9	1.7	0.9
25/LBW/7-M1-(1,2,3)	10.0	10.0	26.9	5.2	2.7
25/LBW/7-M2-(1,2,3)	3.5	3.5	3.1	1.8	0.9
25/LBW/7-M3-(1,2,3)	6.4	6.4	11.0	3.3	1.7
**Sample Series Name**	Confidence Interval of the Standard Deviation (+95.0%)	Coefficient of Variation	Standard Error	Skewness	Standard Error of Skewness
25/LBW/7-R-(1,2,3)	10.7	3.7	1.0	−0.3	1.2
25/LBW/7-M1-(1,2,3)	32.6	5.5	3.0	−1.3	1.2
25/LBW/7-M2-(1,2,3)	11.0	2.0	1.0	−0.3	1.2
25/LBW/7-M3-(1,2,3)	20.8	3.7	1.9	1.2	1.2

## Data Availability

The original contributions presented in this study are included in the article. Further inquiries can be directed to the corresponding author.

## Supplementary Materials

The following supporting information can be downloaded at: https://www.mdpi.com/article/10.3390/ma19061218/s1, Table S1: Mix compositions used for determining the initial and final setting times of cement pastes, including the reference mix (R) and mixes modified with waste-derived materials (M4–M5) [own analysis]; Table S2: Individual values of mechanical strength measurements under static external loading. Characteristic compressive strength (f_ck,cube_) of type “B” cubic specimens for the reference and material-modified series (R, M1, M2, and M3) [own analysis]; Table S3: Frequency table of measured characteristic compressive strength (f_ck,cube_) values for the reference series specimens [own analysis]; Table S4: Frequency table of measured characteristic compressive strength (f_ck,cube_) values for the M1 series specimens [own analysis]; Table S5: Frequency table of measured characteristic compressive strength (f_ck,cube_) values for the M2 series specimens [own analysis]; Table S6: Frequency table of measured characteristic compressive strength (f_ck,cube_) values for the M3 series specimens [own analysis]; Table S7: Individual values of mechanical strength measurements under static external loading. Splitting tensile strength (f_ct_) of type “B” cubic specimens for the reference and modified series material-modified series (R, M1, M2, and M3) [own analysis]; Table S8: Frequency table of measured splitting tensile strength (f_ct_) values for the reference series R specimens [own analysis]; Table S9: Frequency table of measured splitting tensile strength (f_ct_) values for the M1 series specimens [own analysis]; Table S10: Frequency table of measured splitting tensile strength (f_ct_) values for the M2 series specimens [own analysis]; Table S11: Frequency table of measured splitting tensile strength (f_ct_) values for the M3 series specimens [own analysis]; Table S12: Table of descriptive statistics for the measured splitting tensile strength (f_ct_) values of specimens from series R, M1, M2, and M3 [own analysis]; Table S13: Individual bulk density (ρ) measurement values. Bulk density (ρ) of type “B” cubic specimens for the reference and material-modified series (R, M1, M2, and M3) [own analysis]; Table S14: Table of descriptive statistics for the measured bulk density (ρ) values for specimens from series R, M1, M2, and M3 [own analysis]; Table S15: Individual surface water absorption (n_w_) measurement values. Surface water absorption (n_w_) of type “B” cubic specimens for the reference and material-modified series (R, M1, M2, and M3) [own analysis]; Table S16: Table of descriptive statistics for the measured surface water absorption (n_w_) values for specimens from series R, M1, M2, and M3 [own analysis]; Table S17: Table of descriptive statistics for the measured characteristic compressive strength (f_ck,cube_) of specimens from series R, M1, M2 and M3 (internal frost resistance test of concrete prior to 50 freeze–thaw cycles) [own analysis]; Table S18: Table of descriptive statistics for the measured characteristic compressive strength (f_ck,cube_) of specimens from series R, M1, M2 and M3 (internal frost resistance test of concrete prior to 50 freeze–thaw cycles) [own analysis]; Table S19: Table of descriptive statistics for the measured mass of specimens from series R, M1, M2 and M3 (internal freeze–thaw resistance test of concrete prior to 50 F–T cycles) [own analysis]; Table S20: Table of descriptive statistics for the measured changes in mass of specimens from series R, M1, M2 and M3 (internal freeze–thaw resistance test of concrete after 50 F–T cycles) [own analysis]; Table S21: Individual readings of the initial and final setting times of cement paste for specimens from series R and M5 [own analysis]; Table S22: Strength of correlation relationships for the initial and final setting times of cement paste specimens from series R and M5 [own analysis]; Table S23: Strength of correlation relationships for the initial and final setting times of cement paste for samples from series R and M5 (complete correlation results for the analyzed sample series) [own analysis]; Table S24: Table of descriptive statistics for the measured initial setting time of cement paste specimens from series R and M5 [own analysis]; Table S25: Table of descriptive statistics for the measured final setting time of cement paste specimens from series R and M5 [own analysis].
