# Supplementary material for: Eco-Friendly Rapid-Setting Concrete Incorporating Waste-Derived Additives for Post-Disaster Reconstruction
Source: Materials (Basel). 2026 Mar 19;19(6):1218. doi: 10.3390/ma19061218 (PMC13027813; doi:10.3390/ma19061218)
Supplement: Supplementary file 1 [file materials-19-01218-s001.zip › materials-4176715-supplementary.pdf]

SUPPLEMENTARY MATERIALS

Table S1. Mix compositions used for determining the initial and final setting times of cement pastes, including the reference mix (R) and mixes modified with waste-derived materials (M4–M5) [own analysis].

No.	Cement paste component	Component content in cement paste [kg/m³]		
		R (Reference)	M4 (2.50% aqueous sodium silicate solution admixture)	M5 (5.00% aqueous sodium silicate solution admixture)
**1.**	Mixing water (tap water); pH = 7.0	169	169	169
**2.**	Hydraulic binder – special multicomponent cement containing slag and fly ash, type CEM V/A (S-V) 42,5 N-LH/HSR/NA	406	406	406
**3.**	Liquid admixture No. 1 – high-range water-reducing chemical admixture based on an aqueous solution of modified polycarboxylate ethers (dosage: 1.50% by mass of the cementitious binder)	6.10	6.10	6.10
**4.**	Liquid admixture No. 2 – viscosity-modifying chemical admixture based on copolymers (dosage: 1.00% by mass of the cementitious binder)	4.06	4.06	4.06
**5.**	Aqueous sodium silicate solution (polydisperse medium for glass dust and glass powder; dispersing medium)	0	10.16	20.32
**6.**	Soda–lime glass dust, fraction 0.0/0.063 mm (dispersed phase in an aqueous sodium silicate solution)	0	4.05	4.05
**7.**	Soda–lime glass powder, fraction 0.0/0.250 mm (dispersed phase in an aqueous sodium silicate solution)	0	4.05	4.05

Table S2. Individual values of mechanical strength measurements under static external loading. Characteristic compressive strength ($f_{ck,cube}$) of type "B" cubic specimens for the reference and material-modified series (R, M1, M2, and M3) [own analysis].

Sample Name	Cumulative Sample Name	Individual Measurement	Arithmetic Mean	Population Standard Deviation	Standard Error Based on Population Standard Deviation
		[MPa]			
25/LBW/7-R-1	25/LBW/7-R-(1,2,3)	47.1	45.5	1.4	0.8
25/LBW/7-R-2		45.6			
25/LBW/7-R-3		43.7			
25/LBW/7-M1-1	25/LBW/7-M1-(1,2,3)	96.7	95.1	4.2	2.4
25/LBW/7-M1-2		99.3			
25/LBW/7-M1-3		89.3			
25/LBW/7-M2-1	25/LBW/7-M2-(1,2,3)	89.9	89.8	1.4	0.8
25/LBW/7-M2-2		91.5			

25/LBW/7-M2-3		88.0			
25/LBW/7-M3-1	25/LBW/7-M3-(1,2,3)	87.8	90.5	2.7	1.6
25/LBW/7-M3-2		94.2			
25/LBW/7-M3-3		89.5			

The linear relationship, described by the equation y = 12.9800x + 47.7667 (slope coefficient = 12.9800), indicates an increasing trend, with a coefficient of determination R^2 = 0.5179 (R^2 = 51.79%) and an intercept of 47.7667. An increasing trend in the characteristic compressive strength ($f_{ck,cube}$) results was observed for concrete specimens with modified aggregate proportions in hybrid glass–granite aggregate skeletons. The M2 and M3 series exhibited decreases in $f_{ck,cube}$ of (5.3 ± 0.8) MPa and (4.6 ± 1.6) MPa, respectively; for these data, the coefficient of determination (R^2 = 51.79%) represents a model explaining approximately 52% of the variability of the dependent variable (≈52% of the explained variance).

Table S3. Frequency Table of measured characteristic compressive strength ($f_{ck,cube}$) values for the reference series specimens [own analysis].

Frequency Table

Test type: Characteristic compressive strength fck,cube
25/LBW/7-R-(1,2,3); K-S d=0.19785; p>0.20; Lilliefors p>0.20
Shapiro-Wilk W=0.99541, p=0.87047

Class	Count	Cumulative (Count)	Percentage (Valid)	Cumulative Percentage (Valid)	Percentage of Total (Cases)
43.00000<x<=43.50000	0	0	0.0	0.0	0.0
43.50000<x<=44.00000	1	1	33.3	33.3	33.3
44.00000<x<=44.50000	0	1	0.0	33.3	0.0
44.50000<x<=45.00000	0	1	0.0	33.3	0.0
45.00000<x<=45.50000	0	1	0.0	33.3	0.0
45.50000<x<=46.00000	1	2	33.3	66.7	33.3
46.00000<x<=46.50000	0	2	0.0	66.7	0.0
46.50000<x<=47.00000	0	2	0.0	66.7	0.0
47.00000<x<=47.50000	1	3	33.3	100.0	33.3
Missing	0	3	0.0		0.0

Class	Cumulative Percentage (Total)	Expected (Count)	Cumulative (Expected)	Expected Percentage	Cumulative Expected Percentage
43.00000<x<=43.50000	0.0	0.4	0.4	12.4	12.4
43.50000<x<=44.00000	33.3	0.2	0.6	7.0	19.5
44.00000<x<=44.50000	33.3	0.3	0.9	9.1	28.5
44.50000<x<=45.00000	33.3	0.3	1.2	10.7	39.2
45.00000<x<=45.50000	33.3	0.3	1.5	11.6	50.8
45.50000<x<=46.00000	66.7	0.3	1.9	11.5	62.3
46.00000<x<=46.50000	66.7	0.3	2.2	10.5	72.8
46.50000<x<=47.00000	66.7	0.3	2.4	8.8	81.6
47.00000<x<=47.50000	100.0	0.2	2.7	6.8	88.4
Missing	100.0				

Table S3 covers the range of measured characteristic compressive strength ($f_{ck,cube}$) values of 43.0–47.5 MPa. Three individual occurrences were recorded in the following intervals: 43.5–44.0 MPa, 45.5–46.0 MPa, and 47.0–47.5 MPa, each accounting for 33.3% of the valid final results and of the total number of cases. The remaining intervals exhibited zero values (no occurrences of the measured quantity). The occurrence ranges of 88.0–100.0 MPa for the M1 series specimens are presented in Table S4.

Table S4. Frequency Table of measured characteristic compressive strength ($f_{ck,cube}$) values for the M1 series specimens [own analysis].

Frequency Table

Test type: Characteristic compressive strength $f_{ck,cube}$
25/LBW/7-M1-(1,2,3); K-S d=0.28777; p>0.20; Lilliefors p>0.20
Shapiro-Wilk W=0.92868, p=0.48368

Class	Count	Cumulative (Count)	Percentage (Valid)	Cumulative Percentage (Valid)	Percentage of Total (Cases)
88.00000<x<=90.00000	1	1	33.3	33.3	33.3
90.00000<x<=92.00000	0	1	0.0	33.3	0.0
92.00000<x<=94.00000	0	1	0.0	33.3	0.0
94.00000<x<=96.00000	0	1	0.0	33.3	0.0
96.00000<x<=98.00000	1	2	33.3	66.7	33.3
98.00000<x<=100.0000	1	3	33.3	100.0	33.3
Missing	0	3	0.0		0.0

Class	Cumulative Percentage (Total)	Expected (Count)	Cumulative (Expected)	Expected Percentage	Cumulative Expected Percentage
88.00000<x<=90.00000	33.3	0.5	0.5	16.3	16.3
90.00000<x<=92.00000	33.3	0.3	0.8	11.2	27.5
92.00000<x<=94.00000	33.3	0.4	1.2	14.1	41.6
94.00000<x<=96.00000	33.3	0.5	1.7	15.3	56.9
96.00000<x<=98.00000	66.7	0.4	2.1	14.3	71.2
98.00000<x<=100.0000	100.0	0.3	2.5	11.6	82.8
Missing	100.0				

Table S4 covers the range of measured characteristic compressive strength ($f_{ck,cube}$) values of 88.0–100.0 MPa. Three individual occurrences were recorded in the following intervals: 88.0–90.0 MPa, 96.0–98.0 MPa, and 98.0–100.0 MPa, each accounting for 33.3% of the valid final results and of the total number of cases. The remaining intervals exhibited zero values (no occurrences of the measured quantity). The occurrence ranges of 87.5–91.5 MPa for the M2 series specimens are presented in Table S5.

Table S5. Frequency Table of measured characteristic compressive strength ($f_{ck,cube}$) values for the M2 series specimens [own analysis].

Frequency Table					
**Test type: Characteristic compressive strength $f_{ck,cube}$ 25/LBW/7-M2-(1,2,3); K-S d=0,18942; p>0.20; Lilliefors p>0.20 Shapiro-Wilk W=0.99756, p=0.90556.**					
**Class**	**Count**	**Cumulative (Count)**	**Percentage (Valid)**	**Cumulative Percentage (Valid)**	**Percentage of Total (Cases)**
87.50000<x<=88.00000	1	1	33.3	33.3	33.3
88.00000<x<=88.50000	0	1	0.0	33.3	0.0
88.50000<x<=89.00000	0	1	0.0	33.3	0.0
89.00000<x<=89.50000	0	1	0.0	33.3	0.0
89.50000<x<=90.00000	1	2	33.3	66.7	33.3
90.00000<x<=90.50000	0	2	0.0	66.7	0.0
90.50000<x<=91.00000	0	2	0.0	66.7	0.0
91.00000<x<=91.50000	1	3	33.3	100.0	33.3
Missing	0	3	0.0		0.0
Class	**Cumulative Percentage (Total)**	**Expected (Count)**	**Cumulative (Expected)**	**Expected Percentage**	**Cumulative Expected Percentage**
87.50000<x<=88.00000	33.3	0.5	0.5	15.2	15.2
88.00000<x<=88.50000	33.3	0.2	0.7	7.7	22.9
88.50000<x<=89.00000	33.3	0.3	1.0	9.5	32.4
89.00000<x<=89.50000	33.3	0.3	1.3	10.8	43.2
89.50000<x<=90.00000	66.7	0.3	1.6	11.3	54.5
90.00000<x<=90.50000	66.7	0.3	2.0	11.0	65.5
90.50000<x<=91.00000	66.7	0.3	2.3	9.8	75.3
91.00000<x<=91.50000	100.0	0.2	2.5	8.1	83.4
Missing	100.0				

Table S5 covers the range of measured characteristic compressive strength ($f_{ck,cube}$) values of 89.5–91.5 MPa. Three individual occurrences were recorded in the following intervals: 87.5–88.0 MPa, 89.5–90.0 MPa, and 91.0–91.5 MPa, each accounting for 33.3% of the valid final results and of the total number of cases. The remaining intervals exhibited zero values (no occurrences of the measured quantity). The occurrence ranges of 87.0–95.0 MPa for the M3 series specimens are presented in Table S6 .

Table S6. Frequency Table of measured characteristic compressive strength ($f_{ck,cube}$) values for the M3 series specimens [own analysis].

Frequency Table

Test type: Characteristic compressive strength $f_{ck,cube}$
25/LBW/7-M3-(1,2,3); K-S d=0,28521; p>0.20; Lilliefors p>0.20
Shapiro-Wilk W=0.93176, p=0.49522.

Class	Count	Cumulative (Count)	Percentage (Valid)	Cumulative Percentage (Valid)	Percentage of Total (Cases)
87.00000<x<=88.00000	1	1	33.3	33.3	33.3
88.00000<x<=89.00000	0	1	0.0	33.3	0.0
89.00000<x<=90.00000	1	2	33.3	66.7	33.3
90.00000<x<=91.00000	0	2	0.0	66.7	0.0
91.00000<x<=92.00000	0	2	0.0	66.7	0.0
92.00000<x<=93.00000	0	2	0.0	66.7	0.0
93.00000<x<=94.00000	0	2	0.0	66.7	0.0
94.00000<x<=95.00000	1	3	33.3	100.0	33.3
Missing	0	3	0.0		0.0

Class	Cumulative Percentage (Total)	Expected (Count)	Cumulative (Expected)	Expected Percentage	Cumulative Expected Percentage
87.00000<x<=88.00000	33.3	0.7	0.7	22.5	22.5
88.00000<x<=89.00000	33.3	0.3	1.0	10.0	32.5
89.00000<x<=90.00000	66.7	0.3	1.3	11.5	44.0
90.00000<x<=91.00000	66.7	0.4	1.7	12.0	56.0
91.00000<x<=92.00000	66.7	0.3	2.0	11.5	67.5
92.00000<x<=93.00000	66.7	0.3	2.3	10.0	77.5
93.00000<x<=94.00000	66.7	0.2	2.6	8.0	85.4
94.00000<x<=95.00000	100.0	0.2	2.7	5.8	91.3
Missing	100.0				

Table S6 covers the range of measured characteristic compressive strength ($f_{ck,cube}$) values of 87.0–95.0 MPa. Three individual occurrences were recorded in the following intervals: 87.0–88.0 MPa, 89.0–90.0 MPa, and 94.0–95.0 MPa, each accounting for 33.3% of the valid final results and of the total number of cases. The remaining intervals exhibited zero values (no occurrences of the measured quantity).

The measured values of the characteristic compressive strength ($f_{ck,cube}$) were subjected to descriptive statistical analysis, including the median, mode, skewness, mean values (trimmed mean, arithmetic mean, harmonic mean, and Winsorized mean), minimum and maximum values, quantiles (lower and upper quartiles and percentiles), interquartile range, variances, confidence intervals for standard deviations, and standard errors relative to population and sample standard deviations.

Table S7. Individual values of mechanical strength measurements under static external loading. Splitting tensile strength (f_{ct}) of type "B" cubic specimens for the reference and modified series material-modified series (R, M1, M2, and M3) [own analysis].

Sample Name	Cumulative Sample Name	Individual Measurement	Arithmetic Mean	Population Standard Deviation	Standard Error Based on Population Standard Deviation
		[MPa]			
25/LBW/7-R-1	25/LBW/7-R-(1,2,3)	2.10	2.18	0.19	0.11
25/LBW/7-R-2		2.45			
25/LBW/7-R-3		2.00			
25/LBW/7-M1-1	25/LBW/7-M1-(1,2,3)	5.45	4.82	0.51	0.29
25/LBW/7-M1-2		4.80			
25/LBW/7-M1-3		4.20			
25/LBW/7-M2-1	25/LBW/7-M2-(1,2,3)	4.45	3.83	0.91	0.52
25/LBW/7-M2-2		4.50			
25/LBW/7-M2-3		2.55			
25/LBW/7-M3-1	25/LBW/7-M3-(1,2,3)	3.70	4.22	0.48	0.28
25/LBW/7-M3-2		4.10			
25/LBW/7-M3-3		4.85			

The splitting tensile strength (f_{ct}) tests were carried out on four series of standard cubic specimens, with each series comprising three representative specimens. The highest population standard deviation was recorded for the 25/LBW/7-M2 series (0.91 MPa), while the lowest was observed for the reference series 25/LBW/7-R (0.19 MPa), indicating the greatest and least variability, respectively. The lowest individual values of splitting tensile strength (f_{ct}) were recorded for the unmodified reference series 25/LBW/7-R, whereas the highest individual f_{ct} values were observed for the 25/LBW/7-M1 series (arithmetic mean equal to 4.82 ± 0.29 MPa).

Table S8. Frequency Table of measured splitting tensile strength (f_{ct}) values for the reference series R specimens [own analysis].

Frequency Table<br>Test type: Splitting tensile strength (f_{ct})<br>25/LBW/7-R-(1,2,3); K-S d=0.30450; p>0.20; Lilliefors p<0.20<br>Shapiro-Wilk W=0.90672, p=0.40721.					
**Class**	**Count**	**Cumulative (Count)**	**Percentage (Valid)**	**Cumulative Percentage (Valid)**	**Percentage of Total (Cases)**
1.900000<x<=2.000000	1	1	33.33	33.33	33.33
2.000000<x<=2.100000	1	2	33.33	66.67	33.33
2.100000<x<=2.200000	0	2	0.00	66.67	0.00
2.200000<x<=2.300000	0	2	0.00	66.67	0.00
2.300000<x<=2.400000	0	2	0.00	66.67	0.00
2.400000<x<=2.500000	1	3	33.33	100.00	33.33

Missing	0	3	0.00		0.00

Class	Cumulative Percentage (Total)	Expected (Count)	Cumulative (Expected)	Expected Percentage	Cumulative Expected Percentage
1.900000<x<=2.000000	33.33	0.66	0.66	21.89	21.89
2.000000<x<=2.100000	66.67	0.43	1.09	14.33	36.22
2.100000<x<=2.200000	66.67	0.50	1.58	16.59	52.81
2.200000<x<=2.300000	66.67	0.48	2.07	16.11	68.93
2.300000<x<=2.400000	66.67	0.39	2.46	13.12	82.04
2.400000<x<=2.500000	100.00	0.27	2.73	8.95	90.99
Missing	100.00				

The class interval of 1.90–2.50 MPa for the splitting tensile strength (f_{ct}) is presented in Table S10. Three observations were recorded within this range, comprising one occurrence in each of the following classes: 1.90–2.00 MPa, 2.00–2.10 MPa, and 2.40–2.50 MPa. Each class therefore accounted for approximately 33.3% of the total results, with a cumulative frequency of three, corresponding to 100% of the valid observations. The specimens of the 25/LBW/7-R-(1,2,3) series yielded individual splitting tensile strength values assigned to these classes, namely f_{ct} = 2.00 MPa for the 1.90–2.00 MPa interval, fct = 2.10 MPa for the 2.00–2.10 MPa interval, and f_{ct} = 2.45 MPa for the 2.40–2.50 MPa interval. The p-value (where p represents a measure of statistical probability rather than practical probability) was determined as p = 0.40721; therefore, the obtained splitting tensile strength (f_{ct}) results within the above class range were considered statistically non-significant under the frequentist inference framework.

The class interval of 4.00–5.60 MPa for the final splitting tensile strength (f_{ct}) results of the 25/LBW/7-M1 series is presented in Table S9.

Table S9. Frequency Table of measured splitting tensile strength (f_{ct}) values for the M1 series specimens [own analysis].

Frequency Table<br>Test type: Splitting tensile strength (f_{ct})<br>25/LBW/7-M1-(1,2,3); K-S d=.17782; p>0.20; Lilliefors p>0.20<br>Shapiro-Wilk W=0.99947, p=0.95590.					
**Class**	**Count**	**Cumulative (Count)**	**Percentage (Valid)**	**Cumulative Percentage (Valid)**	**Percentage of Total (Cases)**
4.000000<x<=4.200000	1	1	33.33	33.33	33.33
4.200000<x<=4.400000	0	1	0.00	33.33	0.00
4.400000<x<=4.600000	0	1	0.00	33.33	0.00
4.600000<x<=4.800000	1	2	33.33	66.67	33.33
4.800000<x<=5.000000	0	2	0.00	66.67	0.00
5.000000<x<=5.200000	0	2	0.00	66.67	0.00
5.200000<x<=5.400000	0	2	0.00	66.67	0.00
5.400000<x<=5.600000	1	3	33.33	100.00	33.33
Missing	0	3	0.00		0.00
**Class**	**Cumulative Percentage (Total)**	**Expected (Count)**	**Cumulative (Expected)**	**Expected Percentage**	**Cumulative Expected Percentage**

4.000000<x<=4.200000	33.33	0.49	0.49	16.20	16.20
4.200000<x<=4.400000	33.33	0.27	0.76	9.06	25.25
4.400000<x<=4.600000	33.33	0.34	1.09	11.19	36.45
4.600000<x<=4.800000	66.67	0.37	1.47	12.49	48.94
4.800000<x<=5.000000	66.67	0.38	1.85	12.60	61.53
5.000000<x<=5.200000	66.67	0.34	2.19	11.48	73.01
5.200000<x<=5.400000	66.67	0.28	2.47	9.45	82.46
5.400000<x<=5.600000	100.00	0.21	2.68	7.03	89.49
Missing	100.00				

The class interval of 4.00–5.60 MPa for the splitting tensile strength (f_{ct}) is presented in Table S9. Three observations were recorded within this range, comprising one occurrence in each of the following classes: 4.00–4.20 MPa, 4.60–4.80 MPa, and 5.40–5.60 MPa. Each class therefore accounted for approximately 33.3% of the total results, with a cumulative frequency of three, corresponding to 100% of the valid observations. The specimens of the 25/LBW/7-M1-(1,2,3) series yielded individual splitting tensile strength (f_{ct}) values assigned to the following classes: f_{ct} = 4.20 MPa for the (4.00–4.20) MPa class, f_{ct} = 4.80 MPa for the (4.60–4.80) MPa class, and fct = 5.45 MPa for the (5.40–5.60) MPa class (classification of f_{ct} measurement values). The p-value was calculated as p = 0.95590; accordingly, the splitting tensile strength (f_{ct}) results within the corresponding class range were regarded as statistically non-significant under the frequentist inference framework.

The class interval of 2.00–4.50 MPa for the final splitting tensile strength (f_{ct}) results of the 25/LBW/7-M2 series is presented in Table S10.

Table S10. Frequency Table of measured splitting tensile strength (f_{ct}) values for the M2 series specimens [own analysis].

**Frequency Table**					
**Test type: Splitting tensile strength (f_{ct})**<br>**25/LBW/7-M2-(1,2,3); K-S d=0.37712; p>0.20; Lilliefors p<0.10**<br>**Shapiro-Wilk W=0.76922, p=0.04295.**					
**Class**	**Count**	**Cumulative (Count)**	**Percentage (Valid)**	**Cumulative Percentage (Valid)**	**Percentage of Total (Cases)**
2.000000<x<=2.500000	0	0	0.00	0.00	0.00
2.500000<x<=3.000000	1	1	33.33	33.33	33.33
3.000000<x<=3.500000	0	1	0.00	33.33	0.00
3.500000<x<=4.000000	0	1	0.00	33.33	0.00
4.000000<x<=4.500000	2	3	66.67	100.00	66.67
Missing	0	3	0.00		0.00

**Class**	**Cumulative Percentage (Total)**	**Expected (Count)**	**Cumulative (Expected)**	**Expected Percentage**	**Cumulative Expected Percentage**
2.000000<x<=2.500000	0.00	0.35	0.35	11.52	11.52
2.500000<x<=3.000000	33.33	0.33	0.68	11.16	22.67
3.000000<x<=3.500000	33.33	0.47	1.15	15.54	38.21
3.500000<x<=4.000000	33.33	0.53	1.68	17.74	55.96
4.000000<x<=4.500000	100.00	0.50	2.18	16.61	72.56
Missing	100.00				

The class interval of 2.00–4.50 MPa for the splitting tensile strength (f_{ct}) is presented in Table S10. Three observations were recorded within the defined class intervals: one observation in the 2.50–3.00 MPa class and two observations in the 4.00–4.50 MPa class. Each observation accounted for approximately 33.3% of the total dataset, yielding a cumulative frequency of three, corresponding to 100% of the valid cases. The specimens of the 25/LBW/7-M2-(1,2,3) series yielded individual splitting tensile strength (f_{ct}) values assigned to the following classes: f_{ct} = 2.55 MPa for the (2.50–3.20) MPa class, fct = 4.45 and f_{ct} =4.50 MPa for the (4.00–4.50) MPa class (classification of f_{ct} measurement values). The p-value was calculated as p = 0.04295; accordingly, the splitting tensile strength (f_{ct}) results within the corresponding class range were regarded as statistically non-significant under the frequentist inference framework.

The class interval of 3.40–5.00 MPa for the final splitting tensile strength (f_{ct}) results of the 25/LBW/7-M3 series is presented in Table S11.

Table S11. Frequency Table of measured splitting tensile strength (f_{ct}) values for the M3 series specimens [own analysis].

**Frequency Table<br>Test type: Splitting tensile strength (f_{ct})<br>25/LBW/7-M3-(1,2,3); K-S d=0.24586; p>0.20; Lilliefors p>0.20<br>Shapiro-Wilk W=0.97005, p=0.66780.**					
**Class**	**Count**	**Cumulative (Count)**	**Percentage (Valid)**	**Cumulative Percentage (Valid)**	**Percentage of Total (Cases)**
3.400000<x<=3.600000	0	0	0.00	0.00	0.00
3.600000<x<=3.800000	1	1	33.33	33.33	33.33
3.800000<x<=4.000000	0	1	0.00	33.33	0.00
4.000000<x<=4.200000	1	2	33.33	66.67	33.33
4.200000<x<=4.400000	0	2	0.00	66.67	0.00
4.400000<x<=4.600000	0	2	0.00	66.67	0.00
4.600000<x<=4.800000	0	2	0.00	66.67	0.00
4.800000<x<=5.000000	1	3	33.33	100.00	33.33
Missing	0	3	0.00		0.00
**Class**	**Cumulative Percentage (Total)**	**Expected (Count)**	**Cumulative (Expected)**	**Expected Percentage**	**Cumulative Expected Percentage**
3.400000<x<=3.600000	0.00	0.44	0.44	14.54	14.54
3.600000<x<=3.800000	33.33	0.28	0.71	9.23	23.77
3.800000<x<=4.000000	33.33	0.35	1.07	11.76	35.53
4.000000<x<=4.200000	66.67	0.40	1.47	13.33	48.86
4.200000<x<=4.400000	66.67	0.40	1.87	13.46	62.33
4.400000<x<=4.600000	66.67	0.36	2.23	12.10	74.43
4.600000<x<=4.800000	66.67	0.29	2.52	9.69	84.11
4.800000<x<=5.000000	100.00	0.21	2.73	6.90	91.02
Missing	100.00				

The class interval of 3.40–5.00 MPa for the splitting tensile strength (f_{ct}) is presented in Table S11. Three observations were recorded within this range, comprising one occurrence in each of the following classes:

3.60–3.80 MPa, 4.00–4.20 MPa, and 4.80–5.00 MPa. Each class therefore accounted for approximately 33.3% of the total results, with a cumulative frequency of three, corresponding to 100% of the valid observations. The specimens of the 25/LBW/7-M3-(1,2,3) series yielded individual splitting tensile strength (f_{ct}) values assigned to the following classes: f_{ct} = 3.70 MPa for the (3.60–3.80) MPa class, f_{ct} = 4.10 MPa for the (4.00–4.20) MPa class, and f_{ct} = 4.85 MPa for the (4.80–5.00) MPa class (classification of f_{ct} measurement values). The p-value was calculated as p = 0.66780; accordingly, the splitting tensile strength (f_{ct}) results within the corresponding class range were regarded as statistically non-significant under the frequentist inference framework.

Table S12. Table of descriptive statistics for the measured splitting tensile strength (f_{ct}) values of specimens from series R, M1, M2, and M3 [own analysis].

**Descriptive Statistics**					
**Test type: Splitting tensile strength (f_{ct})**					
**Sample Series Name**	**Number of Valid Samples (N)**	**Percentage of Valid Samples (%)**	**Arithmetic Mean**	**Confidence Limit (-95.0%)**	**Confidence Limit (95.0%)**
25/LBW/7-R-(1,2,3)	3	100	2.18	1.60	2.77
25/LBW/7-M1-(1,2,3)	3	100	4.82	3.26	6.37
25/LBW/7-M2-(1,2,3)	3	100	3.83	1.07	6.59
25/LBW/7-M3-(1,2,3)	3	100	4.22	2.77	5.67
**Sample Series Name**	**Trimmed Mean (5.0%)**	**Winsorized Mean (5.0%)**	**Geometric Mean**	**Harmonic Mean**	**Median**
25/LBW/7-R-(1,2,3)	2.18	2.18	2.18	2.17	2.10
25/LBW/7-M1-(1,2,3)	4.82	4.82	4.79	4.76	4.80
25/LBW/7-M2-(1,2,3)	3.83	3.83	3.71	3.58	4.45
25/LBW/7-M3-(1,2,3)	4.22	4.22	4.19	4.16	4.10
**Sample Series Name**	**Mode**	**Frequency of Mode**	**Minimum**	**Maximum**	**Lower Quartile**
25/LBW/7-R-(1,2,3)	Multiple	1	2.00	2.45	2.00
25/LBW/7-M1-(1,2,3)	Multiple	1	4.20	5.45	4.20
25/LBW/7-M2-(1,2,3)	Multiple	1	2.55	4.50	2.55
25/LBW/7-M3-(1,2,3)	Multiple	1	3.70	4.85	3.70
**Sample Series Name**	**Upper Quartile**	**10th Percentile**	**20th Percentile**	**30th Percentile**	**40th Percentile**
25/LBW/7-R-(1,2,3)	2.45	2.00	2.00	2.00	2.10
25/LBW/7-M1-(1,2,3)	5.45	4.20	4.20	4.20	4.80
25/LBW/7-M2-(1,2,3)	4.50	2.55	2.55	2.55	4.45
25/LBW/7-M3-(1,2,3)	4.85	3.70	3.70	3.70	4.10
**Sample Series Name**	**50th Percentile**	**60th Percentile**	**70th Percentile**	**80th Percentile**	**90th Percentile**
25/LBW/7-R-(1,2,3)	2.10	2.10	2.45	2.45	2.45
25/LBW/7-M1-(1,2,3)	4.80	4.80	5.45	5.45	5.45
25/LBW/7-M2-(1,2,3)	4.45	4.45	4.50	4.50	4.50
25/LBW/7-M3-(1,2,3)	4.10	4.10	4.85	4.85	4.85
**Sample Series Name**	**Range**	**Interquartile Range**	**Variance**	**Standard Deviation**	**Confidence Interval of the Standard**

					Deviation (-95.0%)
25/LBW/7-R-(1,2,3)	0.45	0.45	0.06	0.24	0.12
25/LBW/7-M1-(1,2,3)	1.25	1.25	0.39	0.63	0.33
25/LBW/7-M2-(1,2,3)	1.95	1.95	1.24	1.11	0.58
25/LBW/7-M3-(1,2,3)	1.15	1.15	0.34	0.58	0.30

**Sample Series Name**	**Confidence Interval of the Standard Deviation (+95.0%)**	**Coefficient of Variation**	**Standard Error**	**Skewness**	**Standard Error of Skewness**
25/LBW/7-R-(1,2,3)	1.49	10.82	0.14	1.39	1.22
25/LBW/7-M1-(1,2,3)	3.93	12.98	0.36	0.12	1.22
25/LBW/7-M2-(1,2,3)	6.99	29.00	0.64	-1.73	1.22
25/LBW/7-M3-(1,2,3)	3.67	13.85	0.34	0.86	1.22

A Table of descriptive statistics was prepared for the final results of the splitting tensile strength (f_{ct}) tests. The statistical measures were derived from individual readings obtained directly from the measuring device. Table S14 presents the descriptive statistics for specimens from the 25/LBW/7-R, 25/LBW/7-M1, 25/LBW/7-M2, and 25/LBW/7-M3 series. Based on the results reported in the table, the following conclusions were drawn:

- The descriptive statistics were calculated on the basis of three valid representative specimens for each test method, with 100% of the specimens considered valid for the final test results.

- The highest arithmetic mean value ($\bar{X}$) was (4.82 ± 0.29) MPa, while the lowest arithmetic mean value ($\bar{X}$) was (2.18 ± 0.11) MPa.

- The trimmed mean (Tm) was equal to the arithmetic mean ($\bar{X}$).

- For the Winsorized mean, no rejection of extreme (minimum or maximum) values from the dataset of obtained splitting tensile strength (f_{ct}) results was applied. This indicates high sensitivity to errors associated with extreme values and the absence of replacement of extreme minimum and maximum strength values with the nearest neighbouring values.

- The central value (a measure of central tendency robust to ordered extreme values) for an odd number of specimens – the median Me (Mdn., 0.5 quantile, second quartile Q2): the minimum median value was Me = 2.10 MPa, indicating that 50.0% of the ordered splitting tensile strength (f_{ct}) values lie below and above this value; the maximum median value was Me = 4.80 MPa, with 50.0% of the ordered f_{ct} values likewise located below and above this value.

- The mode (D, modal value) was identified as multiple, indicating the absence of a dominant observation of splitting tensile strength (f_{ct}). No f_{ct} value occurred more frequently than others within the dataset (mode frequency equal to 1).

- The coefficient of variation (CV) showed the highest observed variability for the 25/LBW/7-M2 series, with CV = 29.0%, indicating a high degree of dispersion and heterogeneity of f_{ct} results compared with the remaining series (25/LBW/7-R, 25/LBW/7-M1, and 25/LBW/7-M3).

- Distribution asymmetry within the datasets, expressed as skewness of f_{ct} values, revealed negative skewness for the 25/LBW/7-M2 series (a single result with As < 0, indicating a left-skewed distribution), and positive skewness for the 25/LBW/7-R, 25/LBW/7-M1, and 25/LBW/7-M3

series (three results with As > 0, indicating right-skewed distributions). Overall, no symmetric distribution of the final f_{ct} results relative to a hypothetical normal distribution was observed.

Table S13. Individual bulk density (ϱ) measurement values. Bulk density (ϱ) of type "B" cubic specimens for the reference and material-modified series (R, M1, M2, and M3) [own analysis].

Sample Name	Cumulative Sample Name	Individual Measurement	Arithmetic Mean	Population Standard Deviation	Standard Error Based on Population Standard Deviation
		[kg/m³]			
25/LBW/7-R-1	25/LBW/7-R-(1,2,3)	2133	2117	16	9
25/LBW/7-R-2		2122			
25/LBW/7-R-3		2095			
25/LBW/7-M1-1	25/LBW/7-M1-(1,2,3)	2367	2361	14	8
25/LBW/7-M1-2		2374			
25/LBW/7-M1-3		2341			
25/LBW/7-M2-1	25/LBW/7-M2-(1,2,3)	2378	2367	8	5
25/LBW/7-M2-2		2362			
25/LBW/7-M2-3		2360			
25/LBW/7-M3-1	25/LBW/7-M3-(1,2,3)	2372	2378	10	6
25/LBW/7-M3-2		2392			
25/LBW/7-M3-3		2370			

Bulk density (ϱ) measurements were conducted on four series of standard cubic specimens, with each series comprising three representative specimens. The highest population standard deviation was observed for the 25/LBW/7-R series, amounting to 16 kg/m³, whereas the lowest population standard deviation was determined for the 25/LBW/7-M2 series, with a value of 8 kg/m³. The lowest individual bulk density (ϱ) values, within the range of 2117–2122 kg/m³, were recorded for the 25/LBW/7-R specimens, which were not subjected to material modification. In contrast, the highest individual bulk density (ϱ) values were observed for the 25/LBW/7-M3 specimens, falling within the range of 2370–2392 kg/m³.

Table S14. Table of descriptive statistics for the measured bulk density (ϱ) values for specimens from series R, M1, M2, and M3 [own analysis].

Descriptive Statistics					
Test type: Bulk density ϱ					
Sample Series Name	Number of Valid Samples (N)	Percentage of Valid Samples (%)	Arithmetic Mean	Confidence Limit (-95.0%)	Confidence Limit (95.0%)
25/LBW/7-R-(1,2,3)	3	100	2117	2068	2165
25/LBW/7-M1-(1,2,3)	3	100	2361	2317	2404
25/LBW/7-M2-(1,2,3)	3	100	2367	2342	2391
25/LBW/7-M3-(1,2,3)	3	100	2378	2348	2408

Sample Series Name	Trimmed Mean (5.0%)	Winsorized Mean (5.0%)	Geometric Mean	Harmonic Mean	Median
25/LBW/7-R-(1,2,3)	2117	2117	2117	2117	2122
25/LBW/7-M1-(1,2,3)	2361	2361	2361	2361	2367
25/LBW/7-M2-(1,2,3)	2367	2367	2367	2367	2362
25/LBW/7-M3-(1,2,3)	2378	2378	2378	2378	2372

Sample Series Name	Mode	Frequency of Mode	Minimum	Maximum	Lower Quartile
25/LBW/7-R-(1,2,3)	Multiple	1	2095	2133	2095
25/LBW/7-M1-(1,2,3)	Multiple	1	2341	2374	2341
25/LBW/7-M2-(1,2,3)	Multiple	1	2360	2378	2360
25/LBW/7-M3-(1,2,3)	Multiple	1	2370	2392	2370

Sample Series Name	Upper Quartile	10th Percentile	20th Percentile	30th Percentile	40th Percentile
25/LBW/7-R-(1,2,3)	2133	2095	2095	2095	2122
25/LBW/7-M1-(1,2,3)	2374	2341	2341	2341	2367
25/LBW/7-M2-(1,2,3)	2378	2360	2360	2360	2362
25/LBW/7-M3-(1,2,3)	2392	2370	2370	2370	2372

Sample Series Name	50th Percentile	60th Percentile	70th Percentile	80th Percentile	90th Percentile
25/LBW/7-R-(1,2,3)	2122	2122	2133	2133	2133
25/LBW/7-M1-(1,2,3)	2367	2367	2374	2374	2374
25/LBW/7-M2-(1,2,3)	2362	2362	2378	2378	2378
25/LBW/7-M3-(1,2,3)	2372	2372	2392	2392	2392

Sample Series Name	Range	Interquartile Range	Variance	Standard Deviation	Confidence Interval of the Standard Deviation (-95.0%)
25/LBW/7-R-(1,2,3)	38	38	382	20	10
25/LBW/7-M1-(1,2,3)	33	33	302	17	9
25/LBW/7-M2-(1,2,3)	18	18	99	10	5
25/LBW/7-M3-(1,2,3)	22	22	146	12	6

Sample Series Name	Confidence Interval of the Standard Deviation (+95.0%)	Coefficient of Variation	Standard Error	Skewness	Standard Error of Skewness
25/LBW/7-R-(1,2,3)	123	1	11	-1	1
25/LBW/7-M1-(1,2,3)	109	1	10	-1	1
25/LBW/7-M2-(1,2,3)	63	0	6	2	1
25/LBW/7-M3-(1,2,3)	76	1	7	2	1

A Table of descriptive statistics was prepared for the final bulk density (ϱ) test results. The statistical measures were derived from individual readings obtained directly from the measuring device. Table S8 presents the descriptive statistics for specimens from the 25/LBW/7-R, 25/LBW/7-M1, 25/LBW/7-M2, and 25/LBW/7-M3 series. Based on the results reported in the table, the following conclusions were drawn:

- The descriptive statistics were calculated on the basis of three valid representative specimens for each test method, with 100% of the specimens considered valid for the final test results.

- The highest arithmetic mean ($\bar{X}$) bulk density was 2378 ± 6 kg/m^3, while the lowest arithmetic mean ($\bar{X}$) was 2117 ± 9 kg/m^3.

- The trimmed mean (Tm) was equal to the arithmetic mean ($\bar{X}$).

- For the Winsorized mean, no extreme minimum or maximum values were replaced within the set of obtained bulk density (ϱ) values, indicating no treatment of outliers.
As a result, the dataset exhibits high sensitivity to extreme values, since the extreme minimum and maximum bulk density (ϱ) values were not substituted with neighbouring values.

- The central value (a measure of central tendency robust to ordered extreme values) for an odd number of specimens – the median Me (Mdn., 0.5 quantile, second quartile Q2): the minimum median value was Me = 2122 kg/m^3, indicating that 50.0% of the ordered splitting tensile strength (f_{ct}) values lie below and above this value; the maximum median value was Me = 2372 kg/m^3, with 50.0% of the ordered f_{ct} values likewise located below and above this value.

- The mode (D) was identified as multiple, indicating the absence of a dominant bulk density value (ϱ). No single value occurred more frequently than others, and the modal frequency equalled one.

- The coefficient of variation (CV) exhibited its highest observed value of CV = 1% for the 25/LBW/7-R, 25/LBW/7-M1, and 25/LBW/7-M3 series, indicating low variability and high homogeneity of bulk density (ϱ) results.

- Distribution asymmetry, expressed as skewness of bulk density (ϱ) values, revealed negative skewness for the 25/LBW/7-R and 25/LBW/7-M1 series (As < 0; left-skewed distributions) and positive skewness for the 25/LBW/7-M2 and 25/LBW/7-M3 series (As > 0; right-skewed distributions). No symmetric distribution of final bulk density (ϱ) results relative to a hypothetical normal distribution was observed.

Table S15. Individual surface water absorption (nw) measurement values. Surface water absorption (nw) of type "B" cubic specimens for the reference and material-modified series (R, M1, M2, and M3) [own analysis].

Sample Name	Cumulative Sample Name	Individual Measurement	Arithmetic Mean	Population Standard Deviation	Standard Error Based on Population Standard Deviation
		[%]			
**25/LBW/7-R-1**	**25/LBW/7-R-(1,2,3)**	**4.40**	**4.50**	0.08	0.05
25/LBW/7-R-2		4.50			
25/LBW/7-R-3		4.60			
25/LBW/7-M1-1	25/LBW/7-M1-(1,2,3)	3.20	3.17	0.12	0.07
25/LBW/7-M1-2		3.30			
25/LBW/7-M1-3		3.00			
25/LBW/7-M2-1	25/LBW/7-M2-(1,2,3)	3.10	2.73	0.45	0.26
25/LBW/7-M2-2		3.00			
25/LBW/7-M2-3		2.10			
25/LBW/7-M3-1	25/LBW/7-M3-(1,2,3)	3.10	2.97	0.09	0.05
25/LBW/7-M3-2		2.90			
25/LBW/7-M3-3		2.90			

Surface water absorption (n_w) tests were conducted on four series of standard cubic specimens, with each series comprising three representative specimens. The highest population standard deviation was recorded for the 25/LBW/7-M2 series, amounting to 0.45%, whereas the lowest population standard deviation was determined for the 25/LBW/7-R series, with a value of 0.08%. The lowest individual surface water absorption (n_w) values, within the range of 2.90–3.10%, were observed for the 25/LBW/7-M3 specimens subjected to material modification. In contrast, the highest individual surface water absorption (n_w) values were recorded for the 25/LBW/7-R specimens, which were not materially modified, falling within the range of 4.40–4.50%.

Table S16. Table of descriptive statistics for the measured surface water absorption (n_w) values for specimens from series R, M1, M2, and M3 [own analysis].

Descriptive Statistics					
Test type: Surface water absorption n_w					
**Sample Series Name**	**Number of Valid Samples (N)**	**Percentage of Valid Samples (%)**	**Arithmetic Mean**	**Confidence Limit (-95.0%)**	**Confidence Limit (95.0%)**
25/LBW/7-R-(1,2,3)	3	100	4.5	4.3	4.7
25/LBW/7-M1-(1,2,3)	3	100	3.2	2.8	3.5
25/LBW/7-M2-(1,2,3)	3	100	3.1	2.9	3.2
25/LBW/7-M3-(1,2,3)	3	100	3.0	2.7	3.3
**Sample Series Name**	**Trimmed Mean (5.0%)**	**Winsorized Mean (5.0%)**	**Geometric Mean**	**Harmonic Mean**	**Median**

25/LBW/7-R-(1,2,3)	4.5	4.5	4.5	4.5	4.5
25/LBW/7-M1-(1,2,3)	3.2	3.2	3.2	3.2	3.2
25/LBW/7-M2-(1,2,3)	3.1	3.1	3.1	3.1	3.1
25/LBW/7-M3-(1,2,3)	3.0	3.0	3.0	3.0	2.9

**Sample Series Name**	**Mode**	**Frequency of Mode**	**Minimum**	**Maximum**	**Lower Quartile**
25/LBW/7-R-(1,2,3)	Multiple	1	4.4	4.6	4.4
25/LBW/7-M1-(1,2,3)	Multiple	1	3.0	3.3	3.0
25/LBW/7-M2-(1,2,3)	3	2	3.0	3.1	3.0
25/LBW/7-M3-(1,2,3)	3	2	2.9	3.1	2.9

**Sample Series Name**	**Upper Quartile**	**10th Percentile**	**20th Percentile**	**30th Percentile**	**40th Percentile**
25/LBW/7-R-(1,2,3)	4.6	4.4	4.4	4.4	4.5
25/LBW/7-M1-(1,2,3)	3.3	3.0	3.0	3.0	3.2
25/LBW/7-M2-(1,2,3)	3.1	3.0	3.0	3.0	3.1
25/LBW/7-M3-(1,2,3)	3.1	2.9	2.9	2.9	2.9

**Sample Series Name**	**50th Percentile**	**60th Percentile**	**70th Percentile**	**80th Percentile**	**90th Percentile**
25/LBW/7-R-(1,2,3)	4.5	4.5	4.6	4.6	4.6
25/LBW/7-M1-(1,2,3)	3.2	3.2	3.3	3.3	3.3
25/LBW/7-M2-(1,2,3)	3.1	3.1	3.1	3.1	3.1
25/LBW/7-M3-(1,2,3)	2.9	2.9	3.1	3.1	3.1

**Sample Series Name**	**Range**	**Interquartile Range**	**Variance**	**Standard Deviation**	**Confidence Interval of the Standard Deviation (-95.0%)**
25/LBW/7-R-(1,2,3)	0.2	0.2	0.0	0.1	0.1
25/LBW/7-M1-(1,2,3)	0.3	0.3	0.0	0.2	0.1
25/LBW/7-M2-(1,2,3)	0.1	0.1	0.0	0.1	0.0
25/LBW/7-M3-(1,2,3)	0.2	0.2	0.0	0.1	0.1

**Sample Series Name**	**Confidence Interval of the Standard Deviation (+95.0%)**	**Coefficient of Variation**	**Standard Error**	**Skewness**	**Standard Error of Skewness**
25/LBW/7-R-(1,2,3)	0.6	2.2	0.1	0.0	1.2
25/LBW/7-M1-(1,2,3)	1.0	4.8	0.1	-0.9	1.2
25/LBW/7-M2-(1,2,3)	0.4	1.9	0.0	-1.7	1.2
25/LBW/7-M3-(1,2,3)	0.7	3.9	0.1	1.7	1.2

A Table of descriptive statistics was prepared for the final surface water absorption (n_w) test results. The statistical measures were derived from individual readings obtained directly from the measuring device. Table S10 presents the descriptive statistics for specimens from the 25/LBW/7-R, 25/LBW/7-M1, 25/LBW/7-M2, and 25/LBW/7-M3 series. Based on the results reported in the table, the following conclusions were drawn:

- The descriptive statistics were calculated on the basis of three valid representative specimens for each test method, with 100% of the specimens considered valid for the final test results.

- The highest arithmetic mean ($\bar{X}$) surface water absorption was 4.50 ± 0.05%, while the lowest arithmetic mean ($\bar{X}$) was 2.73 ± 0.26%.

- The trimmed mean (Tm) was equal to the arithmetic mean ($\bar{X}$).

- For the Winsorized mean, no extreme minimum or maximum values were replaced within the set of obtained bulk density (ϱ) values, indicating no treatment of outliers.
As a result, the dataset exhibits high sensitivity to extreme values, since the extreme minimum and maximum n_w values were not substituted with neighbouring values.

- The central value (a measure of central tendency robust to ordered extreme values) for an odd number of specimens – the median Me (Mdn., 0.5 quantile, second quartile Q2): the minimum median value was Me = 2.90%, indicating that 50.0% of the ordered surface water absorption (n_w) values lie below and above this value; the maximum median value was Me = 4.50%, with 50.0% of the ordered n_w values likewise located below and above this value.

- The mode (D) (modal value) was identified as multiple, with D = 3 for specimens from the 25/LBW/7-M2 and 25/LBW/7-M3 series. This indicates the absence of a dominant surface water absorption (n_w) value, with no n_w value occurring consistently across the final dataset. Consequently, no prevailing characteristic was observed in the data distribution, with modal frequencies equal to one and two, respectively.

- The coefficient of variation (CV) exhibited its highest observed value of CV = 4.8% for specimens from the 25/LBW/7-M1 series, indicating high variability and heterogeneity of surface water absorption (n_w) results in comparison with the remaining dispersion measures for the 25/LBW/7-R, 25/LBW/7-M2, and 25/LBW/7-M3 series.

- Distribution asymmetry, expressed as skewness of surface water absorption (n_w) values, revealed the occurrence of negative skewness for the 25/LBW/7-M1, 25/LBW/7-M2, and 25/LBW/7-M3 series (three cases with As < 0, indicating left-skewed distributions), whereas zero skewness (As = 0) was observed for the 25/LBW/7-R series (a single case), indicating a symmetric distribution of test results.

Table S17. Table of descriptive statistics for the measured characteristic compressive strength ($f_{ck,cube}$) of specimens from series R, M1, M2 and M3 (internal frost resistance test of concrete prior to 50 freeze–thaw cycles) [own analysis].

**Descriptive Statistics**					
**Test type: Characteristic compressive strength ($f_{ck,cube}$) before freeze–thaw cycles**					
**Sample Series Name**	**Number of Valid Samples (N)**	**Percentage of Valid Samples (%)**	**Arithmetic Mean**	**Confidence Limit (-95.0%)**	**Confidence Limit (95.0%)**
25/LBW/7-R-(1,2,3)	6	100	52.0	50.1	53.8
25/LBW/7-M1-(1,2,3)	6	100	93.7	91.9	95.5
25/LBW/7-M2-(1,2,3)	6	100	90.2	88.1	92.2
25/LBW/7-M3-(1,2,3)	6	100	87.6	81.8	93.5
**Sample Series Name**	**Trimmed Mean (5.0%)**	**Winsorized Mean (5.0%)**	**Geometric Mean**	**Harmonic Mean**	**Median**
25/LBW/7-R-(1,2,3)	52.0	52.0	52.0	51.9	51.5
25/LBW/7-M1-(1,2,3)	93.7	93.7	93.7	93.7	93.8
25/LBW/7-M2-(1,2,3)	90.2	90.2	90.2	90.1	90.2
25/LBW/7-M3-(1,2,3)	87.6	87.6	87.5	87.3	88.0

Sample Series Name	Mode	Frequency of Mode	Minimum	Maximum	Lower Quartile
25/LBW/7-R-(1,2,3)	Multiple	1	49.7	54.2	51.0
25/LBW/7-M1-(1,2,3)	Multiple	1	90.6	95.6	93.5
25/LBW/7-M2-(1,2,3)	Multiple	1	87.5	93.3	89.0
25/LBW/7-M3-(1,2,3)	Multiple	1	80.4	96.0	82.8
**Sample Series Name**	**Upper Quartile**	**10th Percentile**	**20th Percentile**	**30th Percentile**	**40th Percentile**
25/LBW/7-R-(1,2,3)	54.0	49.7	51.0	51.0	51.4
25/LBW/7-M1-(1,2,3)	95.0	90.6	93.5	93.5	93.7
25/LBW/7-M2-(1,2,3)	91.0	87.5	89.0	89.0	90.0
25/LBW/7-M3-(1,2,3)	90.7	80.4	82.8	82.8	87.6
**Sample Series Name**	**50th Percentile**	**60th Percentile**	**70th Percentile**	**80th Percentile**	**90th Percentile**
25/LBW/7-R-(1,2,3)	51.5	51.6	54.0	54.0	54.2
25/LBW/7-M1-(1,2,3)	93.8	93.9	95.0	95.0	95.6
25/LBW/7-M2-(1,2,3)	90.2	90.3	91.0	91.0	93.3
25/LBW/7-M3-(1,2,3)	88.0	88.3	90.7	90.7	96.0
**Sample Series Name**	**Range**	**Interquartile Range**	**Variance**	**Standard Deviation**	**Confidence Interval of the Standard Deviation (-95.0%)**
25/LBW/7-R-(1,2,3)	4.5	3.0	3.1	1.8	1.1
25/LBW/7-M1-(1,2,3)	5.0	1.5	3.0	1.7	1.1
25/LBW/7-M2-(1,2,3)	5.8	2.0	3.8	2.0	1.2
25/LBW/7-M3-(1,2,3)	15.6	7.9	31.1	5.6	3.5
**Sample Series Name**	**Confidence Interval of the Standard Deviation (+95.0%)**	**Coefficient of Variation**	**Standard Error**	**Skewness**	**Standard Error of Skewness**
25/LBW/7-R-(1,2,3)	4.3	3.4	0.7	0.3	0.8
25/LBW/7-M1-(1,2,3)	4.2	1.8	0.7	1.2	0.8
25/LBW/7-M2-(1,2,3)	4.8	2.2	0.8	0.4	0.8
25/LBW/7-M3-(1,2,3)	13.7	6.4	2.3	0.2	0.8
**Sample Series Name**	**Kurtosis**	**Standard Error (Kurtosis)**			
25/LBW/7-R-(1,2,3)	-1.3	1.7			
25/LBW/7-M1-(1,2,3)	2.3	1.7			
25/LBW/7-M2-(1,2,3)	0.9	1.7			
25/LBW/7-M3-(1,2,3)	-0.3	1.7			

A table of descriptive statistics was prepared for the final results of the characteristic compressive strength ($f_{ck,cube}$) tests conducted on standard cubic concrete specimens of type “C” (100 × 100 × 100 mm), as part of the internal frost resistance assessment prior to 50 freeze–thaw (F–T) cycles. Table S11 presents the descriptive statistics for specimen series 25/LBW/7-R, 25/LBW/7-M1, 25/LBW/7-M2 and 25/LBW/7-M3. Based on the results reported in the table, the following conclusions were drawn:

- The descriptive statistics were calculated on the basis of six valid representative specimens for each test method, with 100% of the specimens considered valid for the final test results.

- The highest arithmetic mean value (X) was (93.7 ± 0.6) MPa, while the lowest arithmetic mean value (X) was (52.0 ± 0.7) MPa.

- The trimmed mean (Tm) was equal to the arithmetic mean (X).

- For the Winsorized mean, the extreme minimum and maximum outliers were excluded from the dataset of measured characteristic compressive strength ($f_{ck,cube}$) values, resulting in low susceptibility to errors associated with extreme measured values, achieved through replacement of the extreme minimum and maximum characteristic compressive strength ($f_{ck,cube}$) values with the nearest neighbouring values.

- The central value (a measure of central tendency robust to ordered extreme values) for an even number of specimens was represented by the median Me (Mdn., 0.5 quantile, second quartile Q2): – the minimum median value was Me = 51.5 MPa, with 50.0% of the ordered fck,cube values for series R lying below and above this value; – the maximum median value was Me = 93.8 MPa, with 50.0% of the ordered fck,cube values for series M1 lying below and above this value.

- The mode (D, modal value) was identified as multiple, indicating the absence of a dominant observation of characteristic compressive strength ($f_{ck,cube}$); no single value occurred most frequently within the dataset (mode frequency equal to 1).

- The coefficient of variation (CV) reached its highest value (CV = 6.4%) for specimens from series 25/LBW/7-M3, indicating greater variability and heterogeneity of fck,cube results compared with the remaining series (25/LBW/7-R, 25/LBW/7-M1 and 25/LBW/7-M2).

- Asymmetry of the data distributions, expressed by the skewness of the characteristic compressive strength ($f_{ck,cube}$) values: the occurrence of negative skewness for specimens of series 25/LBW/7-M1 (a single result with As < 0, indicating a left-skewed distribution), and positive skewness for specimens of series 25/LBW/7-R, 25/LBW/7-M2, and 25/LBW/7-M3 (three results with As > 0, indicating right-skewed distributions).

Table S18. Table of descriptive statistics for the measured characteristic compressive strength ($f_{ck,cube}$) of specimens from series R, M1, M2 and M3 (internal frost resistance test of concrete prior to 50 freeze–thaw cycles) [own analysis].

Descriptive Statistics

Test type: Characteristic compressive strength ($f_{ck,cube}$) after 50 freeze–thaw cycles (F50).

Sample Series Name	Number of Valid Samples (N)	Percentage of Valid Samples (%)	Arithmetic Mean	Confidence Limit (-95.0%)	Confidence Limit (95.0%)
25/LBW/7-R-(1,2,3)	6	100	35.4	33.5	37.3
25/LBW/7-M1-(1,2,3)	6	100	90.2	84.3	96.1
25/LBW/7-M2-(1,2,3)	6	100	88.0	84.0	91.9
25/LBW/7-M3-(1,2,3)	6	100	89.9	87.8	92.0

Sample Series Name	Trimmed Mean (5.0%)	Winsorized Mean (5.0%)	Geometric Mean	Harmonic Mean	Median
25/LBW/7-R-(1,2,3)	35.4	35.4	35.3	35.3	34.7
25/LBW/7-M1-(1,2,3)	90.2	90.2	90.1	89.9	92.3
25/LBW/7-M2-(1,2,3)	88.0	88.0	87.9	87.8	87.9
25/LBW/7-M3-(1,2,3)	89.9	89.9	89.9	89.9	90.3

Sample Series Name	Mode	Frequency of Mode	Minimum	Maximum	Lower Quartile
25/LBW/7-R-(1,2,3)	34	2	34.0	38.6	34.0
25/LBW/7-M1-(1,2,3)	Multiple	1	80.8	96.6	86.6
25/LBW/7-M2-(1,2,3)	Multiple	1	82.9	92.6	85.1
25/LBW/7-M3-(1,2,3)	Multiple	1	86.8	92.7	88.7

Sample Series Name	Upper Quartile	10th Percentile	20th Percentile	30th Percentile	40th Percentile
25/LBW/7-R-(1,2,3)	36.4	34.0	34.0	34.0	34.1
25/LBW/7-M1-(1,2,3)	92.9	80.8	86.6	86.6	91.7
25/LBW/7-M2-(1,2,3)	91.5	82.9	85.1	85.1	86.8
25/LBW/7-M3-(1,2,3)	90.8	86.8	88.7	88.7	89.9

Sample Series Name	50th Percentile	60th Percentile	70th Percentile	80th Percentile	90th Percentile
25/LBW/7-R-(1,2,3)	34.7	35.2	36.4	36.4	38.6
25/LBW/7-M1-(1,2,3)	92.3	92.8	92.9	92.9	96.6
25/LBW/7-M2-(1,2,3)	87.9	88.9	91.5	91.5	92.6
25/LBW/7-M3-(1,2,3)	90.3	90.6	90.8	90.8	92.7

Sample Series Name	Range	Interquartile Range	Variance	Standard Deviation	Confidence Interval of the Standard Deviation (-95.0%)
25/LBW/7-R-(1,2,3)	4.6	2.4	3.4	1.8	1.1
25/LBW/7-M1-(1,2,3)	15.8	6.3	31.7	5.6	3.5
25/LBW/7-M2-(1,2,3)	9.7	6.4	14.0	3.7	2.3
25/LBW/7-M3-(1,2,3)	5.9	2.1	4.0	2.0	1.3

Sample Series Name	Confidence Interval of the Standard Deviation (+95.0%)	Coefficient of Variation	Standard Error	Skewness	Standard Error of Skewness
25/LBW/7-R-(1,2,3)	4.5	5.2	0.8	1.3	0.8
25/LBW/7-M1-(1,2,3)	13.8	6.2	2.3	-1.0	0.8

25/LBW/7-M2-(1,2,3)	9.2	4.3	1.5	0.1	0.8
25/LBW/7-M3-(1,2,3)	4.9	2.2	0.8	-0.4	0.8
**Sample Series Name**	**Kurtosis**	**Standard Error (Kurtosis)**			
25/LBW/7-R-(1,2,3)	1.0	1.7			
25/LBW/7-M1-(1,2,3)	0.6	1.7			
25/LBW/7-M2-(1,2,3)	-1.5	1.7			
25/LBW/7-M3-(1,2,3)	0.5	1.7			

A table of descriptive statistics was prepared for the final results of the characteristic compressive strength ($f_{ck,cube}$) tests conducted on standard cubic concrete specimens of type “C” (100 × 100 × 100 mm), as part of the internal frost resistance assessment prior to 50 freeze–thaw (F–T) cycles. This table presents the descriptive statistics for specimen series 25/LBW/7-R, 25/LBW/7-M1, 25/LBW/7-M2 and 25/LBW/7-M3. Based on the results reported in the table, the following conclusions were drawn:

- The descriptive statistics were calculated on the basis of six valid representative specimens for each test method, with 100% of the specimens considered valid for the final test results.

- The highest arithmetic mean value ($\bar{X}$) was (90.2 ± 2.1) MPa, while the lowest arithmetic mean value ($\bar{X}$) was (35.4 ± 0.7) MPa.

- The trimmed mean (Tm) was equal to the arithmetic mean ($\bar{X}$).

- For the Winsorized mean, the extreme minimum and maximum outliers were excluded from the dataset of measured characteristic compressive strength ($f_{ck,cube}$) values, resulting in low susceptibility to errors associated with extreme measured values, achieved through replacement of the extreme minimum and maximum characteristic compressive strength (fck,cube) values with the nearest neighbouring values.

- The central value (a measure of central tendency robust to ordered extreme values) for an even number of specimens was represented by the median Me (Mdn., 0.5 quantile, second quartile Q2): – the minimum median value was Me = 34.7 MPa, with 50.0% of the ordered fck,cube values for series R lying below and above this value; – the maximum median value was Me = 92.3 MPa, with 50.0% of the ordered fck,cube values for series M1 lying below and above this value.

- Mode (D, modal value): the modal value of the final results was D = 34 MPa, indicating the occurrence of a dominant observation for the characteristic compressive strength (fck,cube). The frequency of the mode was 2 for D = 34 MPa, while single occurrences were observed for the remaining multiple modes.

- The coefficient of variation (CV) reached its highest value (CV = 6.2%) for specimens from series 25/LBW/7-M1, indicating greater variability and heterogeneity of fck,cube results compared with the remaining series (25/LBW/7-R, 25/LBW/7-M2 and 25/LBW/7-M3).

- Distribution asymmetry (skewness): negative skewness of the fck,cube values was observed for specimens of series 25/LBW/7-M1, 25/LBW/7-M2, and 25/LBW/7-M3 (three cases with As < 0, indicating a left-skewed distribution of test results), whereas positive skewness was identified for specimens of the 25/LBW/7-R series (a single case with As > 0, corresponding to a right-skewed distribution).

Table S19. Table of descriptive statistics for the measured mass of specimens from series R, M1, M2 and M3 (internal freeze–thaw resistance test of concrete prior to 50 F–T cycles) [own analysis].

Descriptive Statistics					
**Test type: Mass of specimens before freeze–thaw cycles (F50).**					
**Sample Series Name**	**Number of Valid Samples (N)**	**Percentage of Valid Samples (%)**	**Arithmetic Mean**	**Confidence Limit (-95.0%)**	**Confidence Limit (95.0%)**
25/LBW/7-R-(1,2,3)	6	100	2196	2177	2214
25/LBW/7-M1-(1,2,3)	6	100	2336	2290	2383
25/LBW/7-M2-(1,2,3)	6	100	2392	2371	2413
25/LBW/7-M3-(1,2,3)	6	100	2348	2309	2387
**Sample Series Name**	**Trimmed Mean (5.0%)**	**Winsorized Mean (5.0%)**	**Geometric Mean**	**Harmonic Mean**	**Median**
25/LBW/7-R-(1,2,3)	2196	2196	2196	2196	2196
25/LBW/7-M1-(1,2,3)	2336	2336	2336	2336	2342
25/LBW/7-M2-(1,2,3)	2392	2392	2392	2392	2394
25/LBW/7-M3-(1,2,3)	2348	2348	2348	2347	2365
**Sample Series Name**	**Mode**	**Frequency of Mode**	**Minimum**	**Maximum**	**Lower Quartile**
25/LBW/7-R-(1,2,3)	Multiple	1	2175	2219	2178
25/LBW/7-M1-(1,2,3)	Multiple	1	2279	2399	2295
25/LBW/7-M2-(1,2,3)	2390	2	2355	2410	2390
25/LBW/7-M3-(1,2,3)	2365	2	2278	2375	2334
**Sample Series Name**	**Upper Quartile**	**10th Percentile**	**20th Percentile**	**30th Percentile**	**40th Percentile**
25/LBW/7-R-(1,2,3)	2210	2175	2178	2178	2190
25/LBW/7-M1-(1,2,3)	2362	2279	2295	2295	2335
25/LBW/7-M2-(1,2,3)	2409	2355	2390	2390	2390
25/LBW/7-M3-(1,2,3)	2370	2278	2334	2334	2365
**Sample Series Name**	**50th Percentile**	**60th Percentile**	**70th Percentile**	**80th Percentile**	**90th Percentile**
25/LBW/7-R-(1,2,3)	2196	2202	2210	2210	2219
25/LBW/7-M1-(1,2,3)	2342	2348	2362	2362	2399
25/LBW/7-M2-(1,2,3)	2394	2398	2409	2409	2410
25/LBW/7-M3-(1,2,3)	2365	2365	2370	2370	2375
**Sample Series Name**	**Range**	**Interquartile Range**	**Variance**	**Standard Deviation**	**Confidence Interval of the Standard Deviation (-95.0%)**
25/LBW/7-R-(1,2,3)	44	32	312	18	11
25/LBW/7-M1-(1,2,3)	120	67	1944	44	28
25/LBW/7-M2-(1,2,3)	55	19	405	20	13
25/LBW/7-M3-(1,2,3)	97	36	1377	37	23
**Sample Series Name**	**Confidence Interval of the Standard Deviation (+95.0%)**	**Coefficient of Variation**	**Standard Error**	**Skewness**	**Standard Error of Skewness**
25/LBW/7-R-(1,2,3)	43	1	7	0	1

25/LBW/7-M1-(1,2,3)	108	2	18	0	1
25/LBW/7-M2-(1,2,3)	49	1	8	-1	1
25/LBW/7-M3-(1,2,3)	91	2	15	-2	1
**Sample Series Name**	**Kurtosis**	**Standard Error (Kurtosis)**			
25/LBW/7-R-(1,2,3)	-2	2			
25/LBW/7-M1-(1,2,3)	-1	2			
25/LBW/7-M2-(1,2,3)	3	2			
25/LBW/7-M3-(1,2,3)	3	2			

A table of descriptive statistics was prepared for the final mass measurement results of specimens from series R, M1, M2 and M3 (internal freeze–thaw resistance test of concrete prior to 50 freeze–thaw cycles). Table S21 presents the descriptive statistics for specimens from series 25/LBW/7-R, 25/LBW/7-M1, 25/LBW/7-M2 and 25/LBW/7-M3. Based on the results reported in the table, the following conclusions were drawn:

- The descriptive statistics were calculated on the basis of six valid representative specimens for each test method, with 100% of the specimens considered valid for the final test results.

- The highest arithmetic mean ($\bar{X}$) surface water absorption was 2392 ± 8%, while the lowest arithmetic mean ($\bar{X}$) was 2196 ± 7%.

- The trimmed mean (Tm) was equal to the arithmetic mean ($\bar{X}$).

- For the Winsorized mean, extreme minimum and maximum outliers were excluded from the dataset of measured mass values for specimens from series R, M1, M2 and M3, resulting in reduced sensitivity to errors associated with extreme observations through replacement with the nearest neighbouring values.

- The central value (a measure of central tendency robust to ordered extreme values) for an odd number of specimens—the median Me (Mdn., 0.5 quantile, second quartile Q2)—showed a minimum value of Me = 2196 g, indicating that 50.0% of the ordered mass values for specimens from series R lie below and above this value; the maximum median value was Me = 2394 g, with 50.0% of the ordered mass values for specimens from series M2 likewise located below and above this value.

- The mode (D, modal value) was identified as D = 2390 g and D = 2365 g; two dominant mass observations were observed for specimens from series M2 and M3, with a modal frequency of 2 for both values.

- The coefficient of variation (CV) reached a maximum value of CV = 2 for specimens from series 25/LBW/7-M1 and 25/LBW/7-M3, indicating low variability and high homogeneity of specimen mass in series M1 and M3 compared with the remaining datasets (CV values for series 25/LBW/7-R and 25/LBW/7-M2).

- Distribution asymmetry (skewness) of mass values for specimens from series R, M1, M2, and M3, showed negative skewness for specimens from series 25/LBW/7-M2 and 25/LBW/7-M3 (two results with As < 0, indicating left-skewed distributions), and zero skewness for specimens from series 25/LBW/7-R and 25/LBW/7-M1 (two results with As = 0, indicating symmetric distributions).

Table S20. Table of descriptive statistics for the measured changes in mass of specimens from series R, M1, M2 and M3 (internal freeze–thaw resistance test of concrete after 50 F–T cycles) [own analysis].

**Descriptive Statistics**

Test type: Mass of specimens after 50 freeze–thaw cycles (F50).					
**Sample Series Name**	**Number of Valid Samples (N)**	**Percentage of Valid Samples (%)**	**Arithmetic Mean**	**Confidence Limit (-95.0%)**	**Confidence Limit (95.0%)**
25/LBW/7-R-(1,2,3)	6	100	2186	2164	2207
25/LBW/7-M1-(1,2,3)	6	100	2324	2279	2368
25/LBW/7-M2-(1,2,3)	6	100	2367	2345	2390
25/LBW/7-M3-(1,2,3)	6	100	2317	2268	2366
**Sample Series Name**	**Trimmed Mean (5.0%)**	**Winsorized Mean (5.0%)**	**Geometric Mean**	**Harmonic Mean**	**Median**
25/LBW/7-R-(1,2,3)	2186	2186	2186	2186	2184
25/LBW/7-M1-(1,2,3)	2324	2324	2323	2323	2328
25/LBW/7-M2-(1,2,3)	2367	2367	2367	2367	2371
25/LBW/7-M3-(1,2,3)	2317	2317	2317	2317	2337
**Sample Series Name**	**Mode**	**Frequency of Mode**	**Minimum**	**Maximum**	**Lower Quartile**
25/LBW/7-R-(1,2,3)	Multiple	1	2161	2210	2168
25/LBW/7-M1-(1,2,3)	Multiple	1	2266	2391	2293
25/LBW/7-M2-(1,2,3)	Multiple	1	2326	2385	2367
25/LBW/7-M3-(1,2,3)	Multiple	1	2258	2358	2260
**Sample Series Name**	**Upper Quartile**	**10th Percentile**	**20th Percentile**	**30th Percentile**	**40th Percentile**
25/LBW/7-R-(1,2,3)	2207	2161	2168	2168	2178
25/LBW/7-M1-(1,2,3)	2336	2266	2293	2293	2320
25/LBW/7-M2-(1,2,3)	2384	2326	2367	2367	2368
25/LBW/7-M3-(1,2,3)	2355	2258	2260	2260	2324
**Sample Series Name**	**50th Percentile**	**60th Percentile**	**70th Percentile**	**80th Percentile**	**90th Percentile**
25/LBW/7-R-(1,2,3)	2184	2190	2207	2207	2210
25/LBW/7-M1-(1,2,3)	2328	2335	2336	2336	2391
25/LBW/7-M2-(1,2,3)	2371	2373	2384	2384	2385
25/LBW/7-M3-(1,2,3)	2337	2349	2355	2355	2358
**Sample Series Name**	**Range**	**Interquartile Range**	**Variance**	**Standard Deviation**	**Confidence Interval of the Standard Deviation (-95.0%)**
25/LBW/7-R-(1,2,3)	49	39	409	20	13
25/LBW/7-M1-(1,2,3)	125	43	1819	43	27
25/LBW/7-M2-(1,2,3)	59	17	466	22	13
25/LBW/7-M3-(1,2,3)	100	95	2185	47	29
**Sample Series Name**	**Confidence Interval of the Standard Deviation (+95.0%)**	**Coefficient of Variation**	**Standard Error**	**Skewness**	**Standard Error of Skewness**
25/LBW/7-R-(1,2,3)	50	1	8	0	1
25/LBW/7-M1-(1,2,3)	105	2	17	0	1
25/LBW/7-M2-(1,2,3)	53	1	9	-2	1
25/LBW/7-M3-(1,2,3)	115	2	19	-1	1

Sample Series Name	Kurtosis	Standard Error (Kurtosis)
25/LBW/7-R-(1,2,3)	-2	2
25/LBW/7-M1-(1,2,3)	1	2
25/LBW/7-M2-(1,2,3)	4	2
25/LBW/7-M3-(1,2,3)	-2	2

A Table of descriptive statistics was prepared for the final results of mass change measurements for specimens from series R, M1, M2 and M3 (internal freeze–thaw resistance test of concrete after 50 freeze–thaw cycles). Table S22 presents the descriptive statistics for specimens from series 25/LBW/7-R, 25/LBW/7-M1, 25/LBW/7-M2 and 25/LBW/7-M3. Based on the results reported in the table, the following conclusions were drawn:

- The descriptive statistics were calculated on the basis of six valid representative specimens for each test method, with 100% of the specimens considered valid for the final test results.

- The highest arithmetic mean (X̄) surface water absorption was 2367 ± 8%, while the lowest arithmetic mean (X̄) was 2186 ± 8%.

- The trimmed mean (Tm) was equal to the arithmetic mean (X̄).

- For the Winsorized mean, extreme minimum and maximum outliers were excluded from the dataset of measured mass values for specimens from series R, M1, M2 and M3, resulting in reduced sensitivity to errors associated with extreme observations through replacement with the nearest neighbouring values.

- The central value (a measure of central tendency robust to ordered extreme values) for an odd number of specimens—the median Me (Mdn., 0.5 quantile, second quartile Q2)—showed a minimum value of Me = 2186 g, indicating that 50.0% of the ordered mass values for specimens from series R lie below and above this value; the maximum median value was Me = 2367 g, with 50.0% of the ordered mass values for specimens from series M2 likewise located below and above this value.

- The mode (D, modal value) was identified as multiple, indicating the absence of a dominant observation for mass changes in specimens from series R, M1, M2, and M3. No common mass-change value occurred within the dataset (no dominant feature; modal frequency equal to 1).

- The coefficient of variation (CV) reached a maximum value of CV = 2 for specimens from series 25/LBW/7-M1 and 25/LBW/7-M3, indicating low variability and high homogeneity of specimen mass in series M1 and M3 compared with the remaining datasets (CV values for series 25/LBW/7-R and 25/LBW/7-M2).

- Distribution asymmetry (skewness) of mass values for specimens from series R, M1, M2, and M3, showed negative skewness for specimens from series 25/LBW/7-M2 and 25/LBW/7-M3 (two results with As < 0, indicating left-skewed distributions), and zero skewness for specimens from series 25/LBW/7-R and 25/LBW/7-M1 (two results with As = 0, indicating symmetric distributions).

Table S21. Individual readings of the initial and final setting times of cement paste for specimens from series R and M5 [own analysis].

25/LBW/7-R-(1,2,3) - Initial and final setting times

Sample Name	Individual reading – initial setting time	Arithmetic Mean	Population Standard Deviation	Standard Error Based on Population Standard Deviation	Standard error based on the population standard deviation (rounded to the nearest 5 min)
25/LBW/7-R-1	420	380	33	3	5
25/LBW/7-R-2	380				
25/LBW/7-R-3	340				
**Sample Name**	**Individual reading – final setting time**	**Arithmetic Mean**	**Population Standard Deviation**	**Standard Error Based on Population Standard Deviation**	**Standard error based on the population standard deviation (rounded to the nearest 5 min)**
25/LBW/7-R-1	510	497	26	3	5
25/LBW/7-R-2	460				
25/LBW/7-R-3	520				
**25/LBW/7-M5-(1,2,3) - Initial and final setting times**					
**Sample Name**	**Individual reading – initial setting time**	**Arithmetic Mean**	**Population Standard Deviation**	**Standard Error Based on Population Standard Deviation**	**Standard error based on the population standard deviation (rounded to the nearest 5 min)**
25/LBW/7-M5-1	250	213	33	3	5
25/LBW/7-M5-2	220				
25/LBW/7-M5-3	170				
**Sample Name**	**Individual reading – final setting time**	**Arithmetic Mean**	**Population Standard Deviation**	**Standard Error Based on Population Standard Deviation**	**Standard error based on the population standard deviation (rounded to the nearest 5 min)**
25/LBW/7-M5-1	340	307	29	3	5
25/LBW/7-M5-2	310				
25/LBW/7-M5-3	270				

Reference specimens from series 25/LBW/7-R exhibited initial and final setting times of (380 ± 5) min and (497 ± 5) min, respectively, whereas specimens from series 25/LBW/7-M5 showed corresponding setting times of (213 ± 5) min and (307 ± 5) min. A total of three cement paste specimens prepared according to the designed mix formulations were tested for each series, with individual readings reported in Table S15. The standard error values, prior to rounding, were equal to 3 min for all cement paste series. Detailed descriptive statistics for the results of the initial and final setting time tests for the two formulations are presented in Tables 21 and 22.

Calculations were also performed to determine the strength of the correlation relationships between the analysed cement paste series. The results of the correlation analysis are presented in Tables 21 and 22.

Table S22. Strength of correlation relationships for the initial and final setting times of cement paste specimens from series R and M5 [own analysis].

Correlations between cement paste specimen series; correlation coefficients are statistically significant at $p < 0.050$; N = 3.							
**No.**	**Name of cement paste specimen series**	**Arithmetic mean of initial (P) or final (K) setting time**	**Population standard deviation of initial (P) or final (K) setting time**	**25/LBW/7-R-(1,2,3)-P**	**25/LBW/7-R-(1,2,3)-K**	**25/LBW/7-M5-(1,2,3)-P**	**25/LBW/7-M5-(1,2,3)-K**
**1.**	25/LBW/7-R-(1,2,3)-P	380	40	1.000	0.156	0.990	0.997
**2.**	25/LBW/7-R-(1,2,3)-K	497	32	0.156	1.000	0.295	0.236
**3.**	25/LBW/7-M5-(1,2,3)-P	213	40	0.990	0.295	1.000	0.998
**4.**	25/LBW/7-M5-(1,2,3)-K	307	35	0.997	0.236	0.998	1.000

A weak strength of correlation was identified between cement paste specimens prepared according to mix designs 25/LBW/7-R and 25/LBW/7-M5 (r > 0 indicates that an increase in variable X is associated with an increase in variable Y, whereas r < 0 indicates that an increase in X is associated with a decrease in Y). Both negative and positive correlation coefficients were obtained; however, no zero correlation was observed. Despite this, no linear relationship between the analysed variables X and Y was identified (see Table S17 for detailed correlation results).

Detailed results of the calculated correlation relationships (correlations between measured values) for the initial and final setting times of the cement paste are presented in Table S23.

Table S23. Strength of correlation relationships for the initial and final setting times of cement paste for samples from series R and M5 (complete correlation results for the analyzed sample series) [own analysis].

Correlations between cement paste sample series; the calculated correlation coefficients are statistically significant at $p < 0.05$												
**No.**	**Variable X & Variable Y Y**	**Arithmetic Mean**	**Population Standard Deviation**	**r(X,Y)**	**r2**	**t**	**p**	**N – Valid Samples**	**Intercept (dep.: Y)**	**Slope (dep.: Y)**	**Intercept (dep.: X)**	**Slope (dep.: X)**
**1.**	25/LBW/7-R-(1,2,3)-P	380	40									
**2.**	25/LBW/7-R-(1,2,3)-K	497	32	0.156	0.024	0.157	0.901	3	544.167	-0.125	476.129	-0.194
**3.**	25/LBW/7-R-(1,2,3)-P	380	40									
**4.**	25/LBW/7-M5-(1,2,3)-P	213	40	0.990	0.980	6.928	0.091	3	166.667	1.000	171.020	0.980
**5.**	25/LBW/7-R-(1,2,3)-P	380	40									
**6.**	25/LBW/7-M5-(1,2,3)-K	307	35	0.997	0.993	12.124	0.052	3	-25.833	0.875	31.892	1.135
**7.**	25/LBW/7-R-(1,2,3)-K	497	32									
**8.**	25/LBW/7-R-(1,2,3)-P	380	40	0.156	0.024	0.157	0.901	3	476.129	-0.194	544.167	-0.125
**9.**	25/LBW/7-R-(1,2,3)-K	497	32									

**10.**	25/LBW/7-M5-(1,2,3)-P	213	40	0.295	0.087	-0.309	0.809	3	397.581	-0.371	546.735	-0.235
**11.**	25/LBW/7-R-(1,2,3)-K	497	32									
**12.**	25/LBW/7-M5-(1,2,3)-K	307	35	0.236	0.056	-0.243	0.848	3	434.839	-0.258	562.973	-0.216
**13.**	25/LBW/7-M5-(1,2,3)-P	213	40									
**14.**	25/LBW/7-R-(1,2,3)-P	380	40	0.990	0.980	6.928	0.091	3	171.020	0.980	166.667	1.000
**15.**	25/LBW/7-M5-(1,2,3)-P	213	40									
**16.**	25/LBW/7-R-(1,2,3)-K	497	32	0.295	0.087	-0.309	0.809	3	546.735	-0.235	397.581	-0.371
**17.**	25/LBW/7-M5-(1,2,3)-P	213	40									
**18.**	25/LBW/7-M5-(1,2,3)-K	307	35	0.998	0.996	16.358	0.039	3	121.633	0.867	-138.919	1.149
**19.**	25/LBW/7-M5-(1,2,3)-K	307	35									
**20.**	25/LBW/7-R-(1,2,3)-P	380	40	0.997	0.993	12.124	0.052	3	31.892	1.135	-25.833	0.875
**21.**	25/LBW/7-M5-(1,2,3)-K	307	35									
**22.**	25/LBW/7-R-(1,2,3)-K	497	32	0.236	0.056	-0.243	0.848	3	562.973	-0.216	434.839	-0.258
**23.**	25/LBW/7-M5-(1,2,3)-K	307	35									
**24.**	25/LBW/7-M5-(1,2,3)-P	213	40	0.998	0.996	16.358	0.039	3	-138.919	1.149	121.633	0.867

Only two series of cement paste samples were considered in the analysis. Series 25/LBW/7-M4 was excluded due to exceeding the initial and final setting times relative to the reference series 25/LBW/7-R. The calculated correlation relationships were deemed statistically insignificant, owing to the limited number of analysed mix designs, each comprising only three specimens in series 25/LBW/7-R and 25/LBW/7-M5. As a result, no causal relationship can be inferred, and even an identified weak correlation between variables X and Y cannot be regarded as representative or meaningful.

Descriptive statistics for the results of the initial setting time of cement paste are presented in Table S24.

Table S24. Table of descriptive statistics for the measured initial setting time of cement paste specimens from series R and M5 [own analysis].

**Descriptive Statistics**					
**Test type: Initial setting time of cement pastes**					
**Sample Series Name**	**Number of Valid Samples (N)**	**Percentage of Valid Samples (%)**	**Arithmetic Mean**	**Confidence Limit (-95.0%)**	**Confidence Limit (95.0%)**
25/LBW/7-R-(1,2,3)	3	100	380	281	479
25/LBW/7-M5-(1,2,3)	3	100	213	113	314
**Sample Series Name**	**Trimmed Mean (5.0%)**	**Winsorized Mean (5.0%)**	**Geometric Mean**	**Harmonic Mean**	**Median**
25/LBW/7-R-(1,2,3)	380	380	379	377	380
25/LBW/7-M5-(1,2,3)	213	213	211	208	220

Sample Series Name	Mode	Frequency of Mode	Minimum	Maximum	Lower Quartile
25/LBW/7-R-(1,2,3)	Multiple	1	340	420	340
25/LBW/7-M5-(1,2,3)	Multiple	1	170	250	170
**Sample Series Name**	**Upper Quartile**	**10th Percentile**	**20th Percentile**	**30th Percentile**	**40th Percentile**
25/LBW/7-R-(1,2,3)	420	340	340	340	380
25/LBW/7-M5-(1,2,3)	250	170	170	170	220
**Sample Series Name**	**50th Percentile**	**60th Percentile**	**70th Percentile**	**80th Percentile**	**90th Percentile**
25/LBW/7-R-(1,2,3)	380	380	420	420	420
25/LBW/7-M5-(1,2,3)	220	220	250	250	250
**Sample Series Name**	**Range**	**Interquartile Range**	**Variance**	**Standard Deviation**	**Confidence Interval of the Standard Deviation (-95.0%)**
25/LBW/7-R-(1,2,3)	80	80	1600	40	21
25/LBW/7-M5-(1,2,3)	80	80	1633	40	21
**Sample Series Name**	**Confidence Interval of the Standard Deviation (+95.0%)**	**Coefficient of Variation**	**Standard Error**	**Skewness**	**Standard Error of Skewness**
25/LBW/7-R-(1,2,3)	251	11	23	0	1
25/LBW/7-M5-(1,2,3)	254	19	23	-1	1

A Table of descriptive statistics was prepared for the final results of the initial setting time tests of cement paste. Table S18 presents the descriptive statistics for specimens from series 25/LBW/7-R and 25/LBW/7-M5. Based on the results reported in the table, the following conclusions were drawn:

- The descriptive statistics were calculated on the basis of three valid representative specimens for each test method, with 100% of the specimens considered valid for the final test results.

- The highest arithmetic mean ($\bar{X}$) surface water absorption was 380 ± 5%, while the lowest arithmetic mean ($\bar{X}$) was 213 ± 5%.

- The trimmed mean (Tm) was equal to the arithmetic mean ($\bar{X}$).

- For the Winsorized mean, no rejection of extreme (minimum or maximum) values from the dataset of obtained splitting tensile strength (fct) results was applied. This indicates high sensitivity to errors associated with extreme values and the absence of replacement of extreme minimum

- The central value (a measure of central tendency robust to ordered extreme values) for an odd number of specimens – the median Me (Mdn., 0.5 quantile, second quartile Q2): the minimum median value was Me = 220 min, indicating that 50.0% of the ordered initial setting time values of the cement paste lie below and above 220 min; the maximum median value was Me = 380 min, with 50.0% of the ordered initial setting time values likewise located below and above 380 min.

- The mode (D) (modal value): the modal values were multiple for specimens from series 25/LBW/7-R and 25/LBW/7-M5, indicating no dominant observation of the initial setting time; consequently, no single

initial setting time value was prevalent within the dataset (absence of a dominant feature in the data set; mode frequency equal to 1).

- The coefficient of variation (CV): the highest observed variability, CV = 19, was recorded for specimens of series 25/LBW/7-M5, indicating high dispersion and heterogeneity of initial setting time results compared with the remaining dispersion measures (CV for series 25/LBW/7-R).

- Distribution asymmetry within the data sets, expressed by the skewness of the initial setting time values: negative skewness was observed for specimens from series 25/LBW/7-M5 (a single result with As < 0, indicating a left-skewed distribution of test results), whereas zero skewness (As = 0) was identified for specimens from series 25/LBW/7-R, indicating a symmetrical distribution of the results.

Descriptive statistics for the results of the final setting time of cement paste are presented in Table S25.

Table S25. Table of descriptive statistics for the measured final setting time of cement paste specimens from series R and M5 [own analysis].

Descriptive Statistics					
**Test type: Final setting time of cement pastes**					
**Sample Series Name**	**Number of Valid Samples (N)**	**Percentage of Valid Samples (%)**	**Arithmetic Mean**	**Confidence Limit (-95.0%)**	**Confidence Limit (95.0%)**
25/LBW/7-R-(1,2,3)	3	100	497	417	577
25/LBW/7-M5-(1,2,3)	3	100	307	219	394
**Sample Series Name**	**Trimmed Mean (5.0%)**	**Winsorized Mean (5.0%)**	**Geometric Mean**	**Harmonic Mean**	**Median**
25/LBW/7-R-(1,2,3)	497	497	496	495	510
25/LBW/7-M5-(1,2,3)	307	307	305	304	310
**Sample Series Name**	**Mode**	**Frequency of Mode**	**Minimum**	**Maximum**	**Lower Quartile**
25/LBW/7-R-(1,2,3)	Multiple	1	460	520	460
25/LBW/7-M5-(1,2,3)	Multiple	1	270	340	270
**Sample Series Name**	**Upper Quartile**	**10th Percentile**	**20th Percentile**	**30th Percentile**	**40th Percentile**
25/LBW/7-R-(1,2,3)	520	460	460	460	510
25/LBW/7-M5-(1,2,3)	340	270	270	270	310
**Sample Series Name**	**50th Percentile**	**60th Percentile**	**70th Percentile**	**80th Percentile**	**90th Percentile**
25/LBW/7-R-(1,2,3)	510	510	520	520	520
25/LBW/7-M5-(1,2,3)	310	310	340	340	340
**Sample Series Name**	**Range**	**Interquartile Range**	**Variance**	**Standard Deviation**	**Confidence Interval of the Standard Deviation (-95.0%)**
25/LBW/7-R-(1,2,3)	60	60	1033	32	17
25/LBW/7-M5-(1,2,3)	70	70	1233	35	18
**Sample Series Name**	**Confidence Interval of the Standard Deviation (+95.0%)**	**Coefficient of Variation**	**Standard Error**	**Skewness**	**Standard Error of Skewness**
25/LBW/7-R-(1,2,3)	202	6	19	-2	1

25/LBW/7-M5-(1,2,3)	221	11	20	0	1

A Table of descriptive statistics was prepared for the final results of the final setting time tests of cement paste. Table S19 presents the descriptive statistics for specimens from series 25/LBW/7-R and 25/LBW/7-M5. Based on the results reported in the table, the following conclusions were drawn:

- The descriptive statistics were calculated on the basis of three valid representative specimens for each test method, with 100% of the specimens considered valid for the final test results.
- The highest arithmetic mean ($\bar{X}$) surface water absorption was 497 ± 5%, while the lowest arithmetic mean ($\bar{X}$) was 307 ± 5%.
- The trimmed mean (Tm) was equal to the arithmetic mean ($\bar{X}$).
- For the Winsorized mean, no rejection of extreme (minimum or maximum) values from the dataset of obtained splitting tensile strength (fct) results was applied. This indicates high sensitivity to errors associated with extreme values and the absence of replacement of extreme minimum
- The central value (a measure of central tendency robust to ordered extreme values) for an odd number of specimens – the median Me (Mdn., 0.5 quantile, second quartile Q2): the minimum median value was Me = 310 min, indicating that 50.0% of the ordered final setting time values of the cement paste lie below and above 310 min; the maximum median value was Me = 510 min, with 50.0% of the ordered final setting time values likewise located below and above 510 min.
- The mode (D) (modal value): the modal values were multiple for specimens from series 25/LBW/7-R and 25/LBW/7-M5, indicating no dominant observation of the final setting time; consequently, no single final setting time value was prevalent within the dataset (absence of a dominant feature in the data set; mode frequency equal to 1).
- The coefficient of variation (CV): the highest observed variability, CV = 11, was recorded for specimens of series 25/LBW/7-M5, indicating high dispersion and heterogeneity of final setting time results compared with the remaining dispersion measures (CV for series 25/LBW/7-R).
- Distribution asymmetry within the data sets, expressed by the skewness of the final setting time values: negative skewness was observed for specimens from series 25/LBW/7-R (a single result with As < 0, indicating a left-skewed distribution of test results), whereas zero skewness (As = 0) was identified for specimens from series 25/LBW/7-M5, indicating a symmetrical distribution of the results.
